# SAVI, *in silico* generation of billions of easily synthesizable compounds through expert-system type rules

**DOI:** 10.1038/s41597-020-00727-4

**Published:** 2020-11-11

**Authors:** Hitesh Patel, Wolf-Dietrich Ihlenfeldt, Philip N. Judson, Yurii S. Moroz, Yuri Pevzner, Megan L. Peach, Victorien Delannée, Nadya I. Tarasova, Marc C. Nicklaus

**Affiliations:** 1 Computer-Aided Drug Design Group, Chemical Biology Laboratory, Center for Cancer Research, National Cancer Institute, National Institutes of Health, Frederick, MD 21702 USA; 2 Xemistry GmbH, Schwalbenweg 5, D-61479 Glashütten, Germany; 3 Heather Lea, Bland Hill, Norwood, Harrogate, HG3 1TE England; 4 Enamine Ltd, 78 Chervonotkatska Street, Suite 1, Kyiv, 02094, Ukraine and Chemspace LLC, 85 Chervonotkatska Street, Suite 1, Kyiv, 02094 Ukraine; 5 AbbVie, Inc., North Chicago, IL 60064 USA; 6 Basic Science Program, Frederick National Laboratory for Cancer Research, Frederick, MD 21702 USA; 7 Synthetic Biologics and Drug Discovery Group, Laboratory of Cancer Immunometabolism, Center for Cancer Research, National Cancer Institute, National Institutes of Health, Frederick, MD 21702 USA

**Keywords:** Chemical libraries, Cheminformatics, Drug screening

## Abstract

We have made available a database of over 1 billion compounds predicted to be easily synthesizable, called Synthetically Accessible Virtual Inventory (SAVI). They have been created by a set of transforms based on an adaptation and extension of the CHMTRN/PATRAN programming languages describing chemical synthesis expert knowledge, which originally stem from the LHASA project. The chemoinformatics toolkit CACTVS was used to apply a total of 53 transforms to about 150,000 readily available building blocks (enamine.net). Only single-step, two-reactant syntheses were calculated for this database even though the technology can execute multi-step reactions. The possibility to incorporate scoring systems in CHMTRN allowed us to subdivide the database of 1.75 billion compounds in sets according to their predicted synthesizability, with the most-synthesizable class comprising 1.09 billion synthetic products. Properties calculated for all SAVI products show that the database should be well-suited for drug discovery. It is being made publicly available for free download from https://doi.org/10.35115/37n9-5738.

## Abstract (summary)

**Table:** 

Measurement(s)	synthetic accessibility of small molecules • small molecule • Compound
Technology Type(s)	computational modeling technique • in silico
Factor Type(s)	chemical structure

Machine-accessible metadata file describing the reported data: 10.6084/m9.figshare.13020602

## Background & Summary

*In silico* screening of large databases of existing screening samples for the purpose of computer-aided drug design has made significant strides in the recent past, both in terms of the methodologies available and the size and diversity of screening sample collections. Aggregated libraries on the order of 100 million on-the-shelf unique compounds are available in the commercial market^1^. Still, this represents only a microscopically small fraction of the drug-like small-molecule space, estimated to be on the order of 10^21^ to 10^63^ possible structures or even larger^2–4^.

Computational tools have been developed over the past four decades to help the synthetic chemists (and/or their CADD colleagues) find a viable synthetic route for a novel molecule. They can be broadly categorized into two classes: synthesizability estimation^5–13^; and synthetic route prediction (variously called computer assisted synthesis design (CASD), computer-assisted organic synthesis (CAOS), computer-assisted synthesis planning (CASP), or computer-assisted reaction design (CARD))^14–32^. These tools had their heyday during the 1980s and 1990s but subsequently fell out of favor as an approach used in practice, and the entire field went essentially dormant for a good decade until the field experienced a revival of sorts in the 2010s.

Most importantly in our context, however, these approaches were all retrosynthetic in nature, i.e. trying to answer the question *for a given molecule*, “can it be synthesized?” or “how do I make it?” It seemed reasonable to turn this question on its head and instead ask: “what can we easily and cheaply synthesize?” and only then “go fishing” (with all the modern CADD approaches) for bioactive compounds in such a large pool of easy-to-synthesize molecules. The forward-synthetic approach started up nearly as early with tools such as AHMOS, CAMEO, AIPHOS etc.^33–43^. With this approach, one can for example *a priori* limit the number of reaction steps to just one, i.e. the simplest possible chemistry. The central point of SAVI is to avoid any synthetic heroics. Likewise, by giving the task of creating new molecules to the computer, one may reduce anthropogenic biases in chemical reaction choices^44^, thus hopefully covering chemical space better.

Three main components are required to make such an approach successful: (1) A set of highly predictive and richly annotated rules; (2) a significant-size database of reliably available and inexpensive starting materials; (3) a chemoinformatics engine capable of combining (1) and (2) to create a large number of molecules, each annotated with a proposed synthetic route description as well as with predicted properties seen as important in contemporary cutting-edge drug design.

A set of rules was published by Hartenfeller *et al*.^45^, presenting robust organic synthesis reactions, encoded as SMIRKS patterns, that could be useful for *in silico* compound design. SMIRKS patterns, however, do not contain, and cannot easily be annotated with, any algorithmically usable chemistry knowledge for the reaction’s successful application in the laboratory. See below for more discussion of SMIRKS-based approaches. We therefore tapped into the source of synthetic transform knowledge with arguably the richest description of the chemical context for each reaction: the knowledgebase that underlies the computational embodiment of E.J. Corey’s seminal work on retrosynthetic analysis, the program LHASA (Logic and Heuristics Applied to Synthetic Analysis)^14,46–50^. A thorough review of knowledge-based expert systems in chemistry has been recently published^51^.

While LHASA is retrosynthetic, SAVI is strictly forward-synthetic. This implied the task to make LHASA transforms, which are written for retrosynthetic application, work in a forward-synthetic context. (A forward-synthetic application of the LHASA rules, LCOLI, was reported in the early 2000s^52^ but does not seem to have progressed to any widely used tool.)

The active development of the LHASA knowledgebase essentially ceased in the late 1990s. Chemistries such as the Suzuki-Miyaura and Buchwald-Hartwig cross-coupling reactions that are widely used nowadays were thus not represented in the LHASA knowledgebase at the beginning of the SAVI project. We have therefore created novel transforms for such (more) modern chemistry.

After posting for free download an early alpha set (610,492 products) in 2015^53^ and subsequently a beta set of the SAVI database comprising over 283 million structures in 2016, we are presenting here description and analysis of a data set of over 1 billion SAVI products^54^. We point out that SAVI is an ongoing project, i.e. the approach and data described here are a snapshot of its current state.

## Methods

### Transforms

#### Language pair CHMTRN/PATRAN for encoding transforms

The rules are written in the twin programming languages called CHMTRN and PATRAN originally developed in the LHASA project^46,47,55^. CHMTRN is probably best described as a hybrid of FORTRAN style programming with numerous buzz words providing a natural-language-like representation of detailed synthetic chemistry knowledge. It is used together with PATRAN, a chemical pattern description language. CHMTRN/PATRAN surpass other reaction transform descriptions such as SMIRKS in several respects: (1) Structural features that may be important for the reaction but are remote from the reaction center can be described and tested for (such as “a hydroxyl group within two atoms of one of the reaction center atoms”); (2) control and conditional functionality (such as “if… then.. else”, and “for each”) and subroutine usage are possible; (3) tests for structural elements other than atoms and bonds, e.g. physico-chemical properties (such as electrophilic localization energy) can be implemented; (4) scoring systems can be implemented.

The rules can employ a scoring system that is based on molecular structural features, which can either facilitate the reaction (e.g., increase the predicted yield), or impede it. The syntactic elements that increase the transform’s baseline score are the so-called ADD statements, and the SUBTRACT statements as their obvious counterpart. A third, related, syntactic element that is available if the author of a rule deems that structural features would make the reaction entirely unlikely to succeed is the KILL statement, whose meaning and effect is obvious. ADD and SUBTRACT values have traditionally been assigned in increments of five, and typically range from 5 to 30. In spite of their quantitative appearance, they are essentially qualitative human assessments.

We have adopted and extended the CHMTRN language for use in the SAVI project. CHMTRN/PATRAN, originally created for the design of retrosynthetic routes, have been re-implemented for the forward-synthetic SAVI project, but remain able to describe retro-, as well as forward, reactions. For any further explanations of these languages including their detailed syntax, we refer to a recent publication^56^.

#### Existing transform sets

The original LHASA knowledgebase in its entirety comprises about 2,300 transforms. We obtained all transforms from the two organizations that maintain it, the non-profit Lhasa Ltd in the UK (Leeds), and the small company LHASA LLC in the US (Cambridge, MA). The entire set is split roughly into 1,000 basic rules for retrosynthesis planning maintained by the latter company, and 1,300 more-complex rules held, and recently made public^57^, by the former.

While a large number of transforms may give power to a retrosynthetic tool – which after all is intended to provide synthetic route suggestions for *any* molecule a user may submit – this is entirely unnecessary and was in fact undesirable at the inception of SAVI as we were looking for well-established chemistries that are easy, reliable, safe, high-yield etc. We therefore initially chose just over ten transforms from the knowledgebase with an emphasis on ring-forming reactions (Table 1), as well as to provide a test set for implementation of the CHMTRN/PATRAN parser, development of the SAVI algorithms, and initial proof of principle of the feasibility of the entire approach. We used the internal quality annotations in the transforms (such as TYPICAL*YIELD, RELIABILITY, CONDITION*FLEXIBILITY etc.) to filter for overall “good” transforms.

**Table 1:** Transforms initially chosen from existing LHASA knowledgebase.

ID	Name	Ring Forming
**1031**	Paal-Knorr Pyrrole Synthesis	Yes
**1039**	Feist Synthesis of Pyrroles	Yes
**1171**	Hantzsch Thiazole Synthesis	Yes
**1391**	Allene 2 + 2 Cycloaddition	Yes
**1439**	Pyrazoles from Beta Carbonyl Carboxylic Acid Derivatives	Yes
**2201**	Fused Arylpyridines via o-Aminocarbonyls	Yes
**2218**	Tetrazoles from Azide and Nitriles	Yes
**2230**	Phthalazin-1-ones from 2-Acylbenzoic Acids	Yes
**2238**	Fused Aryl(2,3-H/R)Pyridines (Pictet-Spengler)	Yes
**2267**	Sonogashira Coupling	No
**2269**	Kabbe Synthesis of 4-Chromanones	Yes
**2630**	Benzazepin-2-ones by Pictet-Spengler Reaction	Yes
**2684**	Benzo[b]furans from 2-Hydroxyphenyl Acetylenes	Yes

#### New transforms

Due to the age of the existing knowledgebase, it did not contain several named reactions that are widely used nowadays, such as Suzukia-Miyaura Cross-Coupling. We therefore created over fifty novel CHMTRN/PATRAN transforms (Table 2).

**Table 2:** Newly developed transforms.

ID	Name	Ring Forming
**2875**	Copper[I]-catalyzed azide-alkyne cycloaddition	Yes
**6003**	Buchwald-Hartwig Ether Formation	No
**6004**	Suzuki-Miyaura Cross-Coupling (Bromo)	No
**6005**	Suzuki-Miyaura Cross-Coupling (Iodo)	No
**6006**	Suzuki-Miyaura Cross-Coupling (Chloro)	No
**6008**	Suzuki-Miyaura Cross-Coupling with Alkene	No
**6009**	Suzuki-Miyaura Cross-Coupling of Alkenes	No
**6013**	Hiyama Aryl-Alkenyl Cross-Coupling	No
**6014**	Hiyama Non-Aromatic Cross-Coupling	No
**6015**	Hiyama Allyl Cross-Coupling	No
**6016**	Hiyama Carbonylative Cross-Coupling	No
**6017**	Hiyama Cross-Coupling with Arylhydrazine	No
**6022**	Liebeskind-Srogl Thioamide Coupling	No
**6024**	Liebeskind-Srogl Nitrile Formation	No
**6025**	Liebeskind-Srogl Heterocyclic Coupling	No
**6026**	Sulfonamide Schotten-Baumann	No
**6027**	Sulfonamide Schotten-Baumann from Sulfonate	No
**6028**	Sulfonamide Schotten-Baumann from Thiol	No
**6029**	Sulfonamide Schotten-Baumann from Aryl Bromide	No
**6031**	Mitsunobu Reaction	No
**6032**	Mitsunobu carbon-carbon bond formation	No
**6033**	Mitsunobu SN2’ Reaction	No
**6034**	Mitsunobu Imide Reaction	No
**6035**	Mitsunobu Aryl Ether Formation	No
**6036**	Mitsunobu Sulfonamide Reaction	No
**6038**	Ester or Amide or Thiolester Formation	No
**6039**	Williamson Ether Synthesis	No
**6041**	Buchwald-Hartwig Reaction	No
**6043**	Buchwald-Hartwig Reaction	No
**7005**	Benzimidazoles from o-Phenylenediamines	Yes
**7009**	Acylsulfonamide from Sulfonamide and Carboxylic Acid	No
**7013**	Benzimidazoles from o-Phenylenediamines and Aldehydes	Yes
**7014**	Benzimidazoles from o-Phenylenediamines and Aldehydes	Yes
**7015**	Sulfonamide from sulfonic acid and amine	No
**7017**	Sulfonamide alkylation with a cyclic ether	No
**7018**	Sulfonamide acylation	No
**7019**	Wittig Reaction	No
**7020**	Wittig via Methoxy-Ylide	No
**7021**	Horner-Wadsworth-Emmons Olefination	No
**7022**	Chan-Lam coupling	No

We focused on transforms that create novel molecules by making significant new bonds, some of which encode ring-forming reactions. In the SAVI production runs that created the data described here we did not use functional group interchange (FGI) transforms, including the newly written Balz-Schiemann Fluorination (ID 6030) and Nitro Reduction to Primary Amine (ID 6040), which have significant expansion potential, being applicable to 96,314,519 and 89,415,518 of the 1.75 billion SAVI products, respectively. They, and potentially other FGI transforms from the original LHASA transform set, may be used for future broadening of the SAVI database.

The general reaction scheme of SAVI in its current version is thus A + B → C (A, B: reactants; C: product) as we have limited the project to single-step application of transforms.

All newly created transforms have however been coded such that they could directly be used in a retrosynthetic way, i.e. should the LHASA program be reactivated, or a successor retrosynthetic tool be created.

### Chemoinformatics parsing of CHMTRN/PATRAN rules and computation of reactions

While CHMTRN/PATRAN was not publicly documented at the beginning of the project, we received sufficient documentation material from the original providers of the transforms to be able to implement a parser and bytecode interpreter, augmented with additional, connected program logic in the chemoinformatics toolkit CACTVS^58^ (Xemistry GmbH, Glashütten, Germany, https://www.xemistry.com/) for at least a subset of these rules. Details of this work will be published elsewhere. We have now provided a description of the CHMTRN language^56^.

**Fig. 1:**
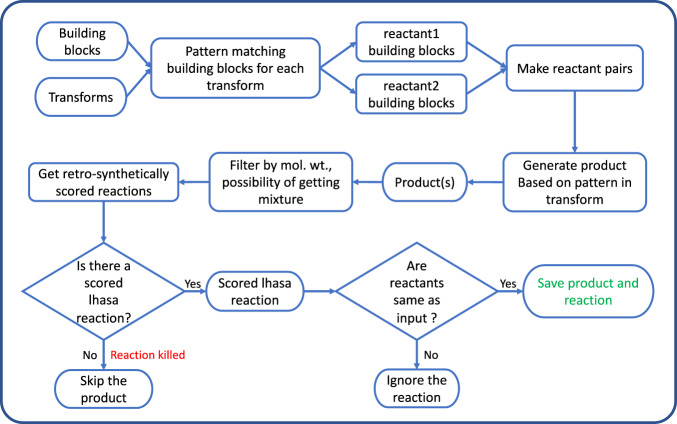
SAVI workflow describing adaptation of retrosynthetic transforms for forward synthesis.

An important aspect of design and implementation of the CHMTRN/PATRAN parser and the SAVI algorithm based on it is that, as already mentioned, the knowledgebase rules were all written for retrosynthetic application, whereas the SAVI project is forward-synthetic. Since we preserved compatibility of newly written transforms with the original retrosynthetic approach, this required a somewhat indirect traversal of the actual rule by first enumerating all possible reactant pairs (if dealing with a two-reactant transform), then testing in a first pass whether the “lhasa react” command in CACTVS produces a possible product, and only then subjecting this (tentative) product to the retrosynthetic analysis of the rule proper (including possibly encountering the above-mentioned ADD, SUBTRACT, or KILL clauses), executed by the “lhasa score” command. This workflow is shown in Fig. 1.

While CACTVS, in an initial transform compilation stage, parses the LHASA transforms written in CHMTRN/PATRAN, the algorithmic contents of the rules are then converted into internal, binary, data structures in CACTVS. The rules are therefore made available on the SAVI download page in both versions: human-readable source code (.src files), and compiled lhasa binary (.clb files).

### Building Blocks (BBs)

Enamine (Kyiv, Ukraine, enamine.net) provided structural details of 155,129 BBs that were in stock as of December 2019. These BBs were standardized to remove fragments and salts. Duplicates were removed via a stereo-sensitive and tautomer-sensitive unique CACTVS hashcode identifier calculated for each building block. Further filters were applied to remove BBs containing less abundant isotopically labelled atoms, metals, as well as structures that were too complex to yield reasonable screening compounds, with the complexity quantitatively defined according to a modified Bertz/Hendrickson algorithm^59–61^. This left us with 152,532 structures. They were used to identify two sets of BBs matching one or the other of the two reactants A and B (see above) for each of the 53 transforms individually, yielding a total of 106 such BB sets. In each of these individual matching procedures, we removed any BB matching both reagent roles (A and B) to avoid forming polymers, as well as any BB matching either one reagent role multiple times at different locations, to avoid forming product mixtures. These filtering steps are obviously specific for each transform and reagent role, since they depend on the required reactive functional groups.

### Protecting groups

Handling protecting groups in the most meaningful way can be somewhat tricky. The issue is that while the planning of a synthetic approach should take protecting groups into account, i.e. present the chemist with a protected product if available, computations on the molecule as a ligand, such as docking, pharmacophore searching, or ADMET property calculations, generally require the unprotected version.

It is possible that a BB set includes the protected version (R1-PG), the unprotected version (R1), or both. The CHMTRN/PATRAN logic considers the effect of exposed or protected functional groups and either rewards or penalizes the reaction accordingly. We therefore did not modify the BBs to computationally add or remove protecting groups. We did however generate modified products by removing protecting groups. Thus, whereas a standard reaction with reactants R1 and R2 yielding product P that does not involve any protecting group is executed to the scheme of:

R1 + R2 →(CHMTRN/PATRAN) P,

if R1 has a protecting group, which produced a product P-PG, we created a deprotected version P:

R1-PG + R2 →(CHMTRN/PATRAN) P-PG →(deprotection) P

This deprotected version is saved in the product set, ready for CADD approaches. The original protected version of the product is added to the SAVI reaction details. In those cases where both a protected and an unprotected version of a building block amenable to a given transform were present in the BB set, a duplicate deprotected product P may have been produced, but only if the unprotected version of the BB did not trigger a KILL statement removing that reaction altogether. Penalization of the reaction with the unprotected BB (if it was not KILLed) is quite likely. It is therefore probable that such reactions are sorted into the “negative” (i.e. penalized) subset of SAVI products (see below) via the classification by reaction scores that we apply.

We used the following structures for the handling of protecting groups:

Amino protecting groups: tert-Butoxy carbamate (Boc), fluorenylmethyloxycarbonyl (Fmoc), benzyloxy carbamate (CBz). Carboxyl protecting groups: tert-Butyl ester (t-Bu ester), benzyl ester (Bz ester). Hydroxyl protecting groups: tert-Butyl ether (t-Bu ether), benzoate (Bz).

### Predicted properties

Each SAVI product has been annotated with over 60 properties, including data about the BBs and proposed reaction (catalog numbers, reactants, general conditions, protection, predicted yield etc.), identifiers/representations of both the BBs and the product, as well as “drug design” properties such as “Rule of Five” (RO5)^62^ and “Rule of Three“^62,63^ violations, PAINS (pan assay interference compounds)^64^ filter matches, FSP3 (fraction of sp^3^ hybridized carbons), and log *P*. The complete list is available on the SAVI Download web page^54^ as well as in sections 1 and 2 of Supplementary Information 1. Section 3 of Supplementary Information 1 shows the fields written in SD file format of a SAVI product file. We are also computing and will make available in the future about 100 different ADME/Tox properties using the program ADMET Predictor (Simulations Plus, Lancaster, CA).

One of the annotations merits a brief elaboration. In addition to the widely used though increasingly controversial^65^ PAINS filter^64^ matches, we have annotated all SAVI products with a score based on 275 rules for identifying potentially reactive or promiscuous compounds that might interfere with biological assays. We believe that these rules, described by Bruns and Watson^66^ as being based on years of assaying experience at Eli Lilly, have more relevance and greater discriminatory and predictive power than the PAINS filters. All 275 rules have been implemented in CACTVS specifically in the context of the SAVI project (with help from Ian Watson), to produce an overall score called “Bruns and Watson demerit” (the lower the value the better).

### Hardware and database

The runs that generated the data presented here were performed in December 2019 – January 2020 on the NIH Biowulf system, a Linux cluster of several tens of thousands of cores (https://hpc.nih.gov/systems/). Due to the “embarrassingly parallel” nature of the SAVI product generation runs (each reactant pair can be processed independently of all others), the entire job was split into nearly 69,000 subjobs, with 4,000 run simultaneously at any time (which was the per-user limit of jobs on Biowulf). The output of the jobs, both the structure data and the annotations, was first written to text files (CSV), then loaded into a PostgreSQL database, which can be queried and analyzed, and whence other formats such as SDF and SMILES lists can be written. A total of about 2,084,000 CPU hours on Biowulf were used to generate this 2020 version of the SAVI database.

## Data Records

### Building blocks used

Out of the total 152,532 accepted Enamine building blocks, application of the pattern-matching part of the 53 productive transforms found 143,365 BBs that fit one or several transforms as a possible reactant (see Online-only Table 1).

### Reactions and unique products generated

**Fig. 2:**
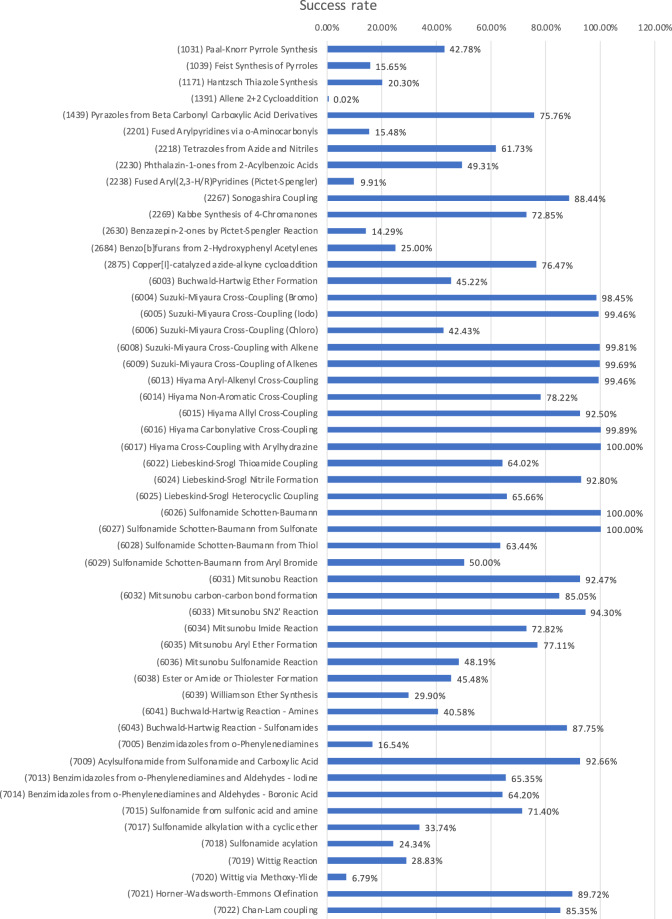
Reaction success rate (percentage of saved reactions out of tested reactant pairs). (Counts were adjusted for duplication in products due to alkene reactivity at both ends of the bond (ID 6009) or tautomerism (IDs 7005, 7013, 7014)).

A total of 3.59 billion reactant pairs were created (Online-only Table 1) and then subjected to the reaction logic of the 53 productive transforms. This yielded 1,748,464,003 reactions saved (Table 3)^54^. Thus, the loss rate caused by encountering KILL statements was about 51%. We re-emphasize that this is a good result: the reduction of the “haystack.” Fig. 2 shows the success rate for each productive transform. The total number of saved reactions per transform is the product of the reaction pair count (Table 3, column 3) with the reaction rate. One can see that the reaction success rates span a range from practically 0% to 100%. It is difficult to decide at this point if these reaction rates are a realistic representation of what actual synthesis would yield for the BBs amenable to each transform or if this indicates that the transforms could still be improved.

**Table 3:** Percentage of total SAVI products and unique molecules saved per scoring class.

Class	SAVI products	Unique within the class	Percentage of total SAVI products
**Plus**	1,094,782,440	976,051,945	62.61%
**Neg0**	609,262	579,532	0.03%
**Neg10**	54,775,204	48,036,148	3.13%
**Neg20**	82,180,372	80,366,188	4.7%
**Neg30**	516,116,725	457,508,945	29.52%
**All combined**	1,748,464,003	1,526,316,392^(a)^	100%

^(a)^The unique-structure numbers for the individual classes do not add up to the unique structures for all classes combined since some products are present in more than one class.

Table 3 shows the numbers of the saved reactions binned into the different scoring classes (“Plus” or “Neg*n*” with *n* equaling at least 0, 10, 20, or 30). We observe that the majority of products (62.6%) are in the Plus class. At the same time, the highest occupancy among the Neg classes is in the highest (i.e. worst) Neg class. This suggests that it may indeed be advisable, especially for the highly productive transforms, to limit oneself to the Plus subsets. The “Scoring Class Distribution” sheet in Supplementary Information 2 shows the scoring class distributions for each individual transform. Two of the transforms, Kabbe Synthesis of 4-Chromanones (ID 2296) and Benzazepin-2-ones by Pictet-Spengler Reaction (ID 2630) generated 10,000 or more products, but none in the Plus class.

As already mentioned, it is entirely possible, and in no way undesirable, that the same molecule is produced by two different reactions, be it from the same building blocks but different procedures, or from different BBs and either the same or different transforms. Counting the unique products out of the 1,748,464,003 saved reactions yielded 1,526,316,392 molecules.

### Success rates and implicit SAR series

If we take the total number of accepted BBs, 152,532, observe that every one of the 53 used reactions essentially follows the pattern A + B → C, we can calculate the theoretically possible maximum number of products as a ½ * 153,532^2^ * 53 ~ 617 billion. (We ignore, for simplicity’s sake, the possibility that in some cases, when multiple reactive groups are present in a BB, one could have A + B → C and B + A → C’. We remove such cases anyway during the reactant pair generation.) Our actually generated product set being 1.75 billion, our success rate in this sense is about 1/350. This reduction is caused by both (a) the fact that most pairs R1 and R2 do not match the PATRAN patterns of any of our transforms, and (b) the 51% loss rate encountered by KILL statements in the CHMTRN reaction logic.

The totality of potential products defined from *N*_*BB*_ building blocks and *n*_*t*_ transforms as *N*_*BB*_^2^ * *n*_*t*_ can be seen as a large, triangular, three-dimensional matrix. Even though this matrix is very sparse, it contains for each filled cell (i.e. saved product) a large set of neighbors with R1 being constant and R2 varying, and vice versa. These sets can be seen as SAR series of sorts, which is a built-in feature of the approach. Due to the variety of chemistries presented in our transforms, the diversity within these series however is likely higher than in typical large-scale combinatorial libraries. Detailed diversity analysis of SAVI will therefore be needed to determine how close these compound series are to SAR series typically used in medicinal chemistry. For each accepted SAVI product, we can estimate the average size of the SAR series as follows. Remembering that the duplication across product space is about 15%, i.e. 85% of the products occur only once across all transforms, we can without too much error project all products onto the flattened two-dimensional matrix sized 143,365 × 143,365, which has 20.6 billion cells. If all cells were filled in a triangular occupation, each generated molecule would have ½ * 143,365 SAR neighbors within each row, and the same number within each column, i.e. a total of about 143,000 SAR neighbors. A SAR neighbor is defined here as a molecule having the same BB R1 but any other R2, and equivalently for R2. However, we have only about 17% of the (triangular) matrix elements filled with truly generated products. This yields an average of about 24,800 SAR neighbors for each SAVI product.

### Protected and unprotected SAVI products

Nearly 10% of the products (153,001,115 products) were generated from at least one protected building block. Protecting groups were removed before writing these products to the SAVI database. A suffix was added to the SAVI ID of a product: UN (UNprotected) if the product was generated from unprotected BBs; DP (DeProtected) if the product was generated from protected BBs but deprotected before writing it to the SAVI database.

## Technical Validation

### Overlap with other databases

We calculated the overlap of SAVI with three large databases (Table 4): the REAL (REadily AccesibLe) database from Enamine^67^, the iResearch Library (iRL) from ChemNavigator/Sigma Aldrich^1^, and PubChem^68^. For PubChem, we measured an overlap rate of 0.3%, i.e. >99% of the SAVI products are not in PubChem. Still, this small percentage corresponds to more than 5 million molecules that are in both databases. Among those are structures that have biological assay data (186,291 compounds). Overlap analysis with DrugBank V.5.1.5^69^ showed that 547 SAVI compounds are in fact drugs. These compounds show that SAVI does generate “real” molecules.

**Table 4:** Overlap of SAVI with other large databases.

Database	Access date	Database size	Overlap with SAVI
REAL^67^	February-2020	~1.2 B	142,806,769
iRL 2017Q4^95^	December-2017	~132 M	10,777,739
PubChem^68^	February-2020	~102 M	5,390,125
SAVI BBs	December-2019	~152 K	34,241

Based on the fact that both the SAVI database and the REAL database use Enamine BBs, it is of interest to know the overlap between those very large databases. We see that on the order of 10% of either database is also present in the other. This is reassuring both in the sense that reasonable chemistry is being created by SAVI and that each of these Enamine-BB-based databases provides its own richness of unique structures.

We also notice that we in fact “re-synthesize” 34,241 of the building blocks themselves. The most likely explanation is that the Enamine BBs contains series of BBs that were synthetically based on each other. This again shows that calling a molecule a building block is mostly a matter of definition and practical considerations, not an invariant chemical property.

### Ring system analysis

As mentioned above, one goal in the creation of the SAVI versions so far has been to build novel molecules, not just modify existing molecules with new or interchanged functional groups. We aimed for this by emphasizing coupling and ring-building transforms. Sixteen of the 53 transforms are exclusively ring-forming (see Tables 1 and 2, third column), which yielded 8,227,198 products with newly formed rings. We note that intra-molecular application of coupling transforms can also lead to the formation of rings. However, this may also lead to polymer formation and was therefore generally excluded in this version of SAVI. Extra information may be added in the future into the transforms themselves to better handle intra-molecular cyclization.

Novel ring systems, i.e. ring systems never before seen in any known compound, have most likely also been generated by SAVI. Conducting a stringent analysis would require a reference body of molecules. Arguably, this would be the Chemical Abstracts Service (CAS) REGISTRY, which is however not readily available in bulk. Manual checking in SciFinder of several hundred cases and extrapolation to the entire database indicate that more than 1,000 novel ring systems may have been created by SAVI.

A count of ring systems, both aromatic and aliphatic, yielded 39,036 unique ring systems in SAVI products. Rings that were already present in the building blocks were also counted. We compared the SAVI ring system count with the ring systems found in three large databases (Table 5).

**Table 5:** Ring systems overlap of SAVI with other large databases.

Database	Access date	Database size	No. of unique ring systems	Overlap with SAVI
REAL^67^	February-2020	~1.2 B	3,389	2,145
iRL 2017Q4^95^	December-2017	~132 M	56,144	2,883
PubChem^68^	February-2020	~102 M	521,946	3,295

We note that the REAL database, while of similar size to SAVI, and based on essentially the same building block set, contain less than a tenth of the number of ring systems found in SAVI. This is likely due to the fact that the chemistries involved in creating SAVI contained more ring-forming transforms than those used for REAL. PubChem, a very diverse database aggregated from hundreds of sources^70^ with very different types of compounds, shows a much larger number of different ring systems. Yet, the iRL, also combining hundreds of sources (but only of screening samples), only slightly surpasses SAVI. Perhaps most interestingly, the ring overlap subsets of SAVI (Table 5) comprised only a few thousand cases for each of the three databases (PubChem: 3,295; REAL: 2,145; iRL: 2,883) while the ring systems present only in SAVI added up to 35,623.

### Distribution of properties relevant for drug design

**Fig. 3:**
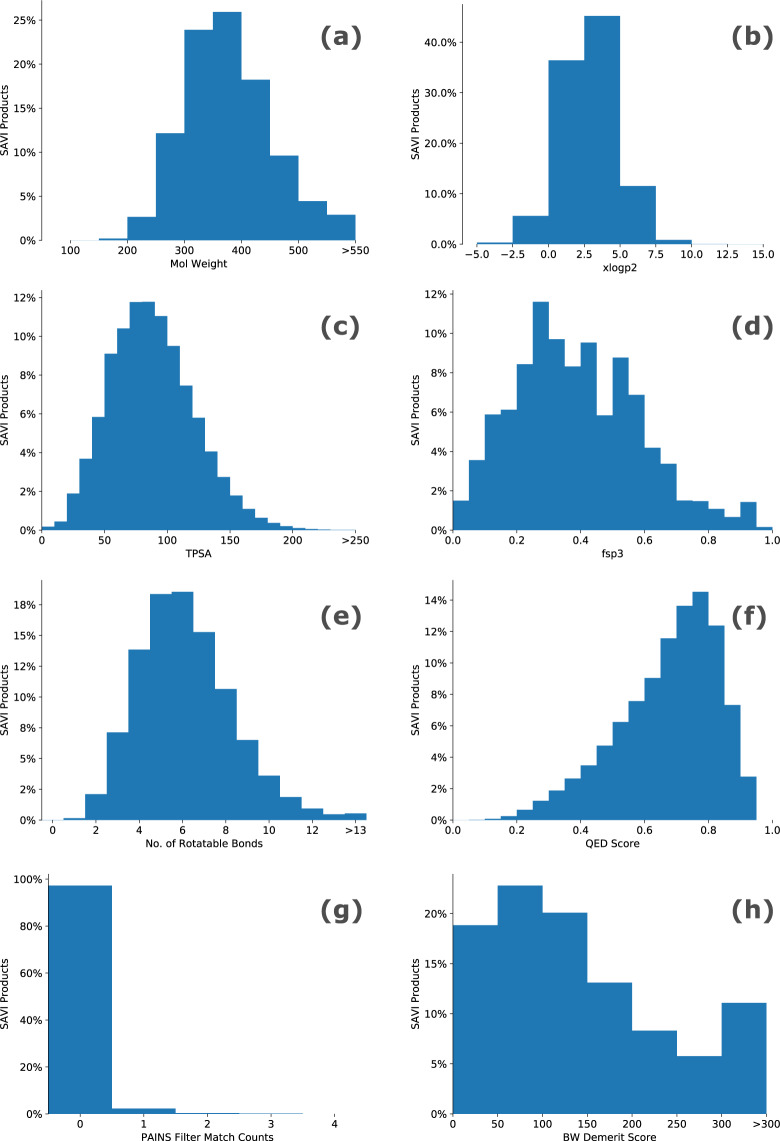
Distributions of drug-design relevant properties calculated for the Plus subset of SAVI (**a**) Molecular weight. (**b**) XlogP2^94^. (**c**) Total Polar Surface Area ($\AA$^2^). (**d**) Fraction of sp^3^ hybridized carbons. (**e**) Number of rotatable bonds. (**f**) QED (Quantitative Estimate of Druglikeness) score^71^. (**g**) PAINS (Pan Assay Interference Compounds) counts. (**h**) Bruns & Watson demerits for Identifying Potentially Reactive or Promiscuous Compounds^66^.

Figure 3 depicts a selection of property distributions of SAVI that are generally seen as important for drug design. The plots shown here are for the Plus subset of SAVI; values for the Neg*n”* sets (plots are provided in sections 5, 6, 7 and 8 of the Supplementary Information 1) show similar distributions. These together with the additional properties provided in section 4 of the Supplementary Information 1 show that the SAVI product set is well suited for drug development. We note that the distribution of QED (quantitative estimate of drug-likeness) values is more drug-like than any of the databases analyzed in the original QED publication^71^. Similarly, the Bruns & Watson demerits^66^ are within the strict limit of <100 used at Eli Lilly in 41% of the Plus SAVI compounds, and within the looser Eli Lilly limit of <160 in 65% of the cases.

### Similarities and differences to other compound generation and synthesis prediction systems

Virtual libraries can significantly enlarge the part of chemistry space amenable to *in silico* screening. Prominent examples of very large libraries of enumerated compounds are the GDB databases, in particular GDB-17 of 166 billion enumerated organic small molecules of up to 17 heavy atoms of C, N, O, S, and halogens^72^. However, such automatically enumerated databases – as well as in principle any purely *de novo* designed molecule – suffer from the significant drawback that no practical synthetic route is *a priori* attached to these structures, and that therefore, in general, (a) manual – and thus expensive – investigation of possible synthetic routes is necessary, (b) resulting routes may be complicated, multi-step syntheses, and (c) synthesis of the molecule may in the end prove altogether unsuccessful (or untenably expensive) even after significant effort.

Pharmaceutical companies have recognized since about 2010 the need for, and benefits of, generating large virtual libraries of easily synthesizable compounds such as Pfizer’s Global Virtual Library^73^, Boehringer Ingleheim’s BI CLAIM^74^, and Eli Lilly’s Proximal Lilly Collection (PLC)^75^, the last probably being closest conceptually to SAVI. Still, there are several, and important, differences between these and SAVI, not least the fact that the resulting virtual libraries are proprietary and thus not available to the public.

The Hartenfeller publication^45^ and its subsequent companion paper analyzing to what degree products generated with these chemistries would cover the bioactivity-relevant chemical space^76^, sparked a number of projects that based large virtual libraries on these SMIRKS-encoded “Hartenfeller reactions”^77–79^. Numerous other projects involving virtual and tangible chemistry spaces and reaction prediction tools have emerged in the recent past^80,81^ and have been reviewed in the literature^82^, as have projects of using such ultra-large libraries for virtual screening^83,84^

The majority of rule-based approaches use SMIRKS to encode the transforms needed to cover the desired chemical space^85,86^. The SMIRKS used by these tools can number in the thousands, especially if retrosynthetic prediction is the goal (“predict the synthesis of a given molecule in any possible way”). SMIRKS, however, do not allow one to directly encode the synthetic chemists’ accumulated knowledge about constraints and limitations of the reactions as a function of the structural details of the reactants. For example, does the SMIRKS for the Sonogashira coupling^45^,

$$[\#6;\,\$({\rm{C}}\,=\,{\rm{C}}\,-\,[\#6]),\$({\rm{c}}:\,{\rm{c}}):\,1][{\rm{Br}},\,{\rm{I}}].\,[{\rm{CH}}1;\,\$({\rm{C}}\#{\rm{CC}}):\,2]\gg [\#6:\,1][{\rm{C}}:\,2]$$

really describe decades of experience of thousands of chemists about when this reaction works, how well, with what yields, and when it might not work at all? On the last point, there is no way to incorporate into a (single) SMIRKS a condition for rejecting the reaction altogether.

SAVI, in contrast, is an expert system approach with a detailed reaction logic that can be incorporated in the CHMTRN/PATRAN files. One such rule can therefore correspond to a large number of SMIRKS (some of which might be quite complicated); and CHMTRN/PATRAN can include features that cannot be expressed in SMIRKS at all (such as computed electron density).

A number of recent approaches are based on statistical evaluation of existing large bodies of reaction data^87–90^ by unleashing modern machine learning methods on these data sets. Molecular structure representation is often done by SMILES. While impressive results have been achieved by these approaches whose central machine-learning algorithms may or may not be aware of chemistry at all, we see several advantages of SAVI compared to these approaches. Learning from existing data sets will always learn what is known, and preferentially learn what is widely used, i.e. strongly represented in the learning set. CHMTRN/PATRAN transforms can, in contrast, be used to add brand-new or unpublished chemistry into SAVI without having to wait for reaction databases to fill up with examples of such reactions. This has not been used much for SAVI up until now because we first wanted to populate the SAVI transform set with reliable, well-known chemistry that would be readily accepted by chemists. However, we have added new transforms in the recent past (not used for creation of the data presented here) as new synthetic approaches are being published. The latest examples include sulfonimidamide synthesis^91^ and modular click chemistry. With accelerating advances in synthetic organic chemistry we expect rapid growth of SAVI^92^.

The usage of sophisticated transforms that incorporate a scoring system makes it possible to use negative outcomes of the reaction logic (KILLed reactions, reactions with SUBTRACT demerits) to create large sets of (computationally) failed reactions, which may be useful for, e.g., machine learning approaches. Such efforts are currently being investigated.

### Multi-step reactions

Multi-step reactions are trivial to conceive in SAVI but daunting in their prospective sizes. For example, taking just the output of the click chemistry transform (transform ID 2875, Copper[I]-catalyzed azide-alkyne cycloaddition), which produced 1 million molecules, as input for a second step (i.e. combining them with the standard BB compounds), yielded more than 50 billion reactant pairs. Taking the entire 1 billion current SAVI output set instead as new BBs can be estimated to yield 1 trillion actually accepted reactions. Techniques such as targeted growing into this huge space of 3-reactant, 2-step, SAVI syntheses will be needed, which will be the topic of future reports.

### Applications

The SAVI database is being used in a number of drug discovery projects at the National Cancer Institute and with collaborators world-wide, including against SARS-CoV-2 targets. Reports on these projects will be published separately.

## Usage Notes

In the context of the SAVI project, we employ a chemoinformatics usage of terms, which may differ from synthetic chemists’ conventions. The (typically: named) chemistries used in SAVI are described by “transforms” (also called “rules”), whereas the application of a transform to a specific set of starting materials yields a “reaction.” For example, there is one Sonogashira coupling transform/rule, but its application to all possible starting materials may yield tens of millions of Sonogashira reactions, each with a specific reaction product. The starting materials are taken from a set of possible reactants, which are also called building blocks (BB(s)). Some of the newly added named reactions were encoded in several different transforms expressing variants of reaction mechanisms, which we call “chemistries.” For example, the Suzuki-Miyaura chemistry is encoded in six different transforms: Suzuki-Miyaura Cross-Coupling (Bromo), Suzuki-Miyaura Cross-Coupling (Iodo), etc. (see Table 2).

## Supplementary information

Supplementary Information 1

Supplementary Information 2

## Data Availability

The academic version of the chemoinformatics toolkit CACTVS is available for free download from https://www.xemistry.com/academic/ for evaluation and for use in research and education (a paid license is required for commercial use). The transforms used in the generation of the SAVI database are freely available from https://cactus.nci.nih.gov/download/savi_download/. The source code of the “lhasa” command in CACTVS that was developed for the SAVI project can be obtained from W.-D. Ihlenfeldt (info@xemistry.com, +49 6174 201455) upon request. Development of a different, more public, way of using CHMTRN/PATRAN transforms for SAVI-type product generation based on open-source code has begun but is in its early stages^93^.

## Online-only Table

**Online-only Table 1:** Reactants found and reactant pairs generated for each transform.

ID	Name	R1	R2	Pairs
**1031**	Paal-Knorr Pyrrole Synthesis	38,317	4	153,268
**1039**	Feist Synthesis of Pyrroles	167	55	9,185
**1171**	Hantzsch Thiazole Synthesis	505	920	464,600
**1391**	Allene 2 + 2 Cycloaddition	17	7,792	132,464
**1439**	Pyrazoles from Beta Carbonyl Carboxylic Acid Derivatives	33	1,691	55,803
**2201**	Fused Arylpyridines via o-Aminocarbonyls	17,257	218	3,762,026
**2218**	Tetrazoles from Azide and Nitriles	7,089	1	7,089
**2230**	Phthalazin-1-ones from 2-Acylbenzoic Acids	55	1,690	92,950
**2238**	Fused Aryl(2,3-H/R)Pyridines (Pictet-Spengler)	1,309	14,095	18,450,355
**2267**	Sonogashira Coupling	1,200	22,839	27,406,800
**2269**	Kabbe Synthesis of 4-Chromanones	5,750	35	201,250
**2630**	Benzazepin-2-ones by Pictet-Spengler Reaction	14	5,092	71,288
**2684**	Benzo[b]furans from 2-Hydroxyphenyl Acetylenes	12	314	3,768
**2875**	Copper[I]-catalyzed azide-alkyne cycloaddition	960	1,646	1,580,160
**6003**	Buchwald-Hartwig Ether Formation	14,834	6,519	96,702,846
**6004**	Suzuki-Miyaura Cross-Coupling (Bromo)	10,857	543	5,895,351
**6005**	Suzuki-Miyaura Cross-Coupling (Iodo)	1,490	543	809,070
**6006**	Suzuki-Miyaura Cross-Coupling (Chloro)	13,115	534	7,003,410
**6008**	Suzuki-Miyaura Cross-Coupling with Alkene	543	91	49,413
**6009**	Suzuki-Miyaura Cross-Coupling of Alkenes	88	5,150	453,200
**6013**	Hiyama Aryl-Alkenyl Cross-Coupling	1,491	2	2,982
**6014**	Hiyama Non-Aromatic Cross-Coupling	76	151	11,476
**6015**	Hiyama Allyl Cross-Coupling	2	80	160
**6016**	Hiyama Carbonylative Cross-Coupling	12,039	2	24,078
**6017**	Hiyama Cross-Coupling with Arylhydrazine	553	2	1,106
**6022**	Liebeskind-Srogl Thioamide Coupling	164	543	89,052
**6024**	Liebeskind-Srogl Nitrile Formation	1	583	583
**6025**	Liebeskind-Srogl Heterocyclic Coupling	330	539	177,870
**6026**	Sulfonamide Schotten-Baumann	1,981	62,784	124,375,104
**6027**	Sulfonamide Schotten-Baumann from Sulfonate	62,994	108	6,803,352
**6028**	Sulfonamide Schotten-Baumann from Thiol	62,491	2,313	144,541,683
**6029**	Sulfonamide Schotten-Baumann from Aryl Bromide	59,213	7,159	423,905,867
**6031**	Mitsunobu Reaction	28,743	5,860	168,433,980
**6032**	Mitsunobu carbon-carbon bond formation	11,858	18	213,444
**6033**	Mitsunobu SN2’ Reaction	29,672	3	89,016
**6034**	Mitsunobu Imide Reaction	6,073	6,146	37,324,658
**6035**	Mitsunobu Aryl Ether Formation	11,848	4,631	54,868,088
**6036**	Mitsunobu Sulfonamide Reaction	10,317	2,130	21,975,210
**6038**	Ester or Amide or Thiolester Formation	38,104	21,136	805,366,144
**6039**	Williamson Ether Synthesis	14,159	24,371	345,068,989
**6041**	Buchwald-Hartwig Reaction - Amines	17,755	36,712	651,821,560
**6043**	Buchwald-Hartwig Reaction - Sulfonamides	10,619	3,516	37,336,404
**7005**	Benzimidazoles from o-Phenylenediamines	190	29,659	5,635,210
**7009**	Acylsulfonamide from Sulfonamide and Carboxylic Acid	1,690	29,508	49,868,520
**7013**	Benzimidazoles from o-Phenylenediamines and Aldehydes - Iodine	346	5,092	1,761,832
**7014**	Benzimidazoles from o-Phenylenediamines and Aldehydes - Boronic Acid	142	5,092	723,064
**7015**	Sulfonamide from sulfonic acid and amine	62,983	108	6,802,164
**7017**	Sulfonamide alkylation with a cyclic ether	1,475	7,500	11,062,500
**7018**	Sulfonamide acylation	3,930	314	1,234,020
**7019**	Wittig Reaction	8,435	58,616	494,425,960
**7020**	Wittig via Methoxy-Ylide	12	14,175	170,100
**7021**	Horner-Wadsworth-Emmons Olefination	5,070	7	35,490
**7022**	Chan-Lam coupling	521	58,891	30,682,211
				3,588,136,173
