# Supplementary material for: SAVI, in silico generation of billions of easily synthesizable compounds through expert-system type rules
Source: Sci Data. 2020 Nov 11;7:384. doi: 10.1038/s41597-020-00727-4 (PMC7658252; doi:10.1038/s41597-020-00727-4)
Supplement: Supplementary file 1 — Supplementary Information 1 [file 41597_2020_727_MOESM1_ESM.pdf]

Supplementary Information for: "SAVI, in silico generation of billions of easily synthesizable compounds through expert-system type rules"

Hitesh Patel[1], Wolf-Dietrich Ihlenfeldt[2], Philip N. Judson[3], Yurii Moroz[4], Yuri Pevzner[1,5], Megan L. Peach[6], Victorien Delannée[1], Nadya Tarasova[7], Marc C. Nicklaus[1]

[1]Computer-Aided Drug Design Group, Chemical Biology Laboratory, Center for Cancer Research, National Cancer Institute, National Institutes of Health, Frederick, MD 21702, USA.

[2]Xemistry GmbH, Schwalbenweg 5, D-61479 Glashütten, Germany.

[3]Heather Lea, Bland Hill, Norwood, Harrogate HG3 1TE, England.

[4]Enamine Ltd, 78 Chervonotkatska Street, Suite 1, Kyiv, 02094, Ukraine and Chemspace LLC, 85 Chervonotkatska Street, Suite 1, Kyiv, 02094, Ukraine

[5]AbbVie, Inc., North Chicago, IL 60064, USA

[6]Basic Science Program, Frederick National Laboratory for Cancer Research, Frederick, MD 21702

[7]Synthetic Biologics and Drug Discovery Group, Laboratory of Cancer Immunometabolism, Center for Cancer Research, National Cancer Institute, National Institutes of Health, Frederick, MD 21702, USA.

Table of Contents

1. Products Properties List 3

2. Reactions Properties List 4

3. Fields of SD file of a SAVI product 6

4. Additional plots of property distribution for “Plus” set 9

5. Plots of property distribution for “Neg0” set 11

6. Plots of property distribution for “Neg10” set 14

7. Plots of property distribution for “Neg20” set 17

8. Plots of property distribution for “Neg30” set 20

1. Products Properties List

SAVI_ID - Product name, SAVI ID
HASHCODE - Product Hashcode
SMILES - Product SMILES
FORMULA - Molecular formula
WEIGHT - Molecular weight
NROTBONDS - Number of rotatable bonds
NHDONORS - Number of H-bond donors
NHACCEPTORS - Number of H-bond acceptors
TPSA - Total polar surface area
FSP3 - Fraction of sp3-hybridized carbons
COMPLEXITY - Complexity (see: W. D. Ihlenfeldt, Ph.D. Thesis, TU Munich, Germany, 1991)
CHEMICAL_BEAUTY - QED score of the product
BENZENOID_INDEX - Benzenoid index
CHARGED_GROUP_COUNTS - Number of charged groups
HYDROGEN_BOND_CENTER_COUNT - Number of hydrogen bond centers
HEAVY_ATOM_COUNT - Number of heavy atoms
STEREO_COUNT - Number of stereo centers
RINGSYSTEM_COUNT - No. of ring systems
NESSSR - Number of extended smallest set of smallest rings (ESSSR)
NSSSR - Number of smallest set of smallest rings (SSSR)
ARORING_COUNT - No. of aromatic rings
ALIRING_COUNT - No. of aliphatic rings
STDINCHI - Standard InChI
STDINCHIKEY - Standard InChI-Key
STEREO_TAUTO_HASH - tautomer-insensitive hashcode
RULE_OF_5_VIOLATIONS - Number of Lipinski "rule of 5" violations
RULE_OF_3_VIOLATIONS - Number of "rule of 3 violations"
SAVI_PAINS_FILTER_MATCH_COUNT - PAINS Filter Match
SAVI_PAINS_FILTER_MATCH_NAME - Name of the PAINS filter match
GENOTOXIC_ALERTS - Genotoxic alerts (see: CE&N Sep 27, 2010, p16ff)
XLOGP2 - Xlogp2
XLOGP - Xlogp
SAVI_BRUNS_WATSON_DEMERIT_SCORE - Bruns and Watson Demerit score (see Bruns, R.F., Watson, I.A. Rules for identifying potentially reactive or promiscuous compounds. J. Med. Chem. 2012, 55,9763-9772)
SAVI_BRUNS_WATSON_DEMERIT_COMPONENTS - Breakdown of Bruns and Watson Demerits

2. Reactions Properties List

SAVI_PROPOSED_TRANSFORM - Name of the reaction

SAVI_PROPOSED_TRANSFORM_ID - ID of the reaction (LHASA ID of the transform that describes this reaction)

SAVI_REACTION_HASHCODE - Hashcode of the reaction

SAVI_REACTION_SMILES - Atom-mapped SMILES of the reaction

SAVI_LHASA_SCORE - "Quality" score of this reaction according to LHASA transform scoring scheme

SAVI_LHASA_SCORING_DELTAPOINTS - Score penalties or rewards given to the reaction

SAVI_LHASA_SCORING_CLASS - Scoring class of the reaction according to highest delta points

SAVI_REACTION_CONDITIONS - Predicted reaction conditions according to LHASA transform rules

SAVI_REACTION_ACTUAL_CONDITIONS - Reaction conditions according to LHASA transform rules

SAVI_REACTION_WARNINGS - Reaction warnings according to LHASA transform rules

SAVI_SSSR_COUNT_CHANGE - SSSR count in product minus combined SSSR count in building blocks

SAVI_ALIRING_COUNT_CHANGE - No. of aliphatic rings changes in reaction

SAVI_ARORING_COUNT_CHANGE - No. of aromatic rings changes in reaction

SAVI_RINCHI - RInChI of the reaction

SAVI_LONG_RINCHIKEY - Long-RInChIKey of the reaction

SAVI_SHORT_RINCHIKEY - Short-RInChIKey of the reaction

SAVI_WEB_RINCHIKEY - Web-RInChIKey of the reaction

SAVI_BUILDING_BLOCK_A_HASHISY - Hashcode of building block A

SAVI_BUILDING_BLOCK_A_SMILES - SMILES of building block A

SAVI_BUILDING_BLOCK_A_BBSOURCE - Source with version of building block A (ENAMINE2019Q4 for this whole SAVI)

SAVI_BUILDING_BLOCK_A_STRID - Enamine catalog ID of building block A

SAVI_BUILDING_BLOCK_A_INCHI - InChI of building block A

SAVI_BUILDING_BLOCK_A_INCHIKEY - InChIKey of building block A

SAVI_BUILDING_BLOCK_A_PROTECTION_NEEDED - Indicates whether protection of reagent A is required in this reaction

SAVI_BUILDING_BLOCK_A_PROTECTED - Indicates whether building block A used in this reaction is already a protected version of the required reagent

SAVI_BUILDING_BLOCK_A_ORDER_LINK - URL of the Enamine cataglog web page for building block A

SAVI_BUILDING_BLOCK_B_HASHISY - Hashcode of building block B

SAVI_BUILDING_BLOCK_B_SMILES - SMILES of building block B

SAVI_BUILDING_BLOCK_B_BBSOURCE - Source with version of building block B (ENAMINE2019Q4 for this whole SAVI)

SAVI_BUILDING_BLOCK_B_STRID - Enamine catalog ID of building block B

SAVI_BUILDING_BLOCK_B_INCHI - InChI of building block B

SAVI_BUILDING_BLOCK_B_INCHIKEY - InChIKey of building block B

SAVI_BUILDING_BLOCK_B_PROTECTION_NEEDED - Indicates whether protection of reagent B is required in this reaction

SAVI_BUILDING_BLOCK_B_PROTECTED - Indicates whether building block B used in this reaction is already a protected version of the required reagent

SAVI_BUILDING_BLOCK_B_ORDER_LINK - URL of the Enamine cataglog web page for building block B

3. Fields of SD file of a SAVI product

> <E_SAVI_ID>

> <E_SMILES>

> <E_HASHISY>

> <E_FORMULA>

> <E_WEIGHT>

> <E_NROTBONDS>

> <E_NHDONORS>

> <E_NHACCEPTORS>

> <E_SAVI_PROTECTED>

> <E_TPSA>

> <E_FSP3>

> <E_COMPLEXITY>

> <E_CHEMICAL_BEAUTY>

> <E_BENZENOID_INDEX>

> <E_CHARGED_GROUP_COUNTS>

> <E_HYDROGEN_BOND_CENTER_COUNT>

> <E_HEAVY_ATOM_COUNT>

> <E_STEREO_COUNT>

> <E_RINGSYSTEM_COUNT>

> <E_NESSSR>

> <E_NSSSR>

> <E_ALIRING_COUNT>

> <E_ARORING_COUNT>

> <E_SAVI_PAINS_FILTER_MATCH_COUNT>

> <E_SAVI_PAINS_FILTER_MATCH_NAME>

> <E_RULE_OF_5_VIOLATIONS>

> <E_GENOTOXIC_ALERTS>

> <E_XLOGP2>

> <E_STEREO_TAUTO_HASH>

> <E_SAVI_BRUNS_WATSON_DEMERIT_SCORE>

> <E_SAVI_BRUNS_WATSON_DEMERIT_COMPONENTS>

> <E_SAVI_PROPOSED_TRANSFORM>

> <E_SAVI_PROPOSED_TRANSFORM_ID>

> <E_SAVI_REACTION_HASHCODE>

> <E_SAVI_REACTION_SMILES>

> <E_SAVI_LHASA_SCORE>

> <E_SAVI_LHASA_SCORING_DELTAPOINTS>

> <E_SAVI_LHASA_SCORING_CLASS>

> <E_SAVI_REACTION_CONDITIONS>

> <E_SAVI_REACTION_ACTUAL_CONDITIONS>

> <E_SAVI_REACTION_WARNINGS>

> <E_SAVI_SSSR_COUNT_CHANGE>

> <E_SAVI_ALIRING_COUNT_CHANGE>

> <E_SAVI_ARORING_COUNT_CHANGE>

> <E_SAVI_RINCHI>

> <E_SAVI_LONG_RINCHIKEY>

> <E_SAVI_SHORT_RINCHIKEY>

> <E_SAVI_WEB_RINCHIKEY>

> <E_SAVI_BUILDING_BLOCK_A_HASHISY>

> <E_SAVI_BUILDING_BLOCK_A_SMILES>

> <E_SAVI_BUILDING_BLOCK_A_BBSOURCE>

> <E_SAVI_BUILDING_BLOCK_A_STRID>

> <E_SAVI_BUILDING_BLOCK_A_INCHI>

> <E_SAVI_BUILDING_BLOCK_A_INCHIKEY>

> <E_SAVI_BUILDING_BLOCK_A_PROTECTION_NEEDED>

> <E_SAVI_BUILDING_BLOCK_A_PROTECTED>

> <E_SAVI_BUILDING_BLOCK_A_ORDER_LINK>

> <E_SAVI_BUILDING_BLOCK_B_HASHISY>

> <E_SAVI_BUILDING_BLOCK_B_SMILES>

> <E_SAVI_BUILDING_BLOCK_B_BBSOURCE>

> <E_SAVI_BUILDING_BLOCK_B_STRID>

> <E_SAVI_BUILDING_BLOCK_B_INCHI>

> <E_SAVI_BUILDING_BLOCK_B_INCHIKEY>

> <E_SAVI_BUILDING_BLOCK_B_PROTECTION_NEEDED>

> <E_SAVI_BUILDING_BLOCK_B_PROTECTED>

> <E_SAVI_BUILDING_BLOCK_B_ORDER_LINK>

4. Additional plots of property distribution for “Plus” set

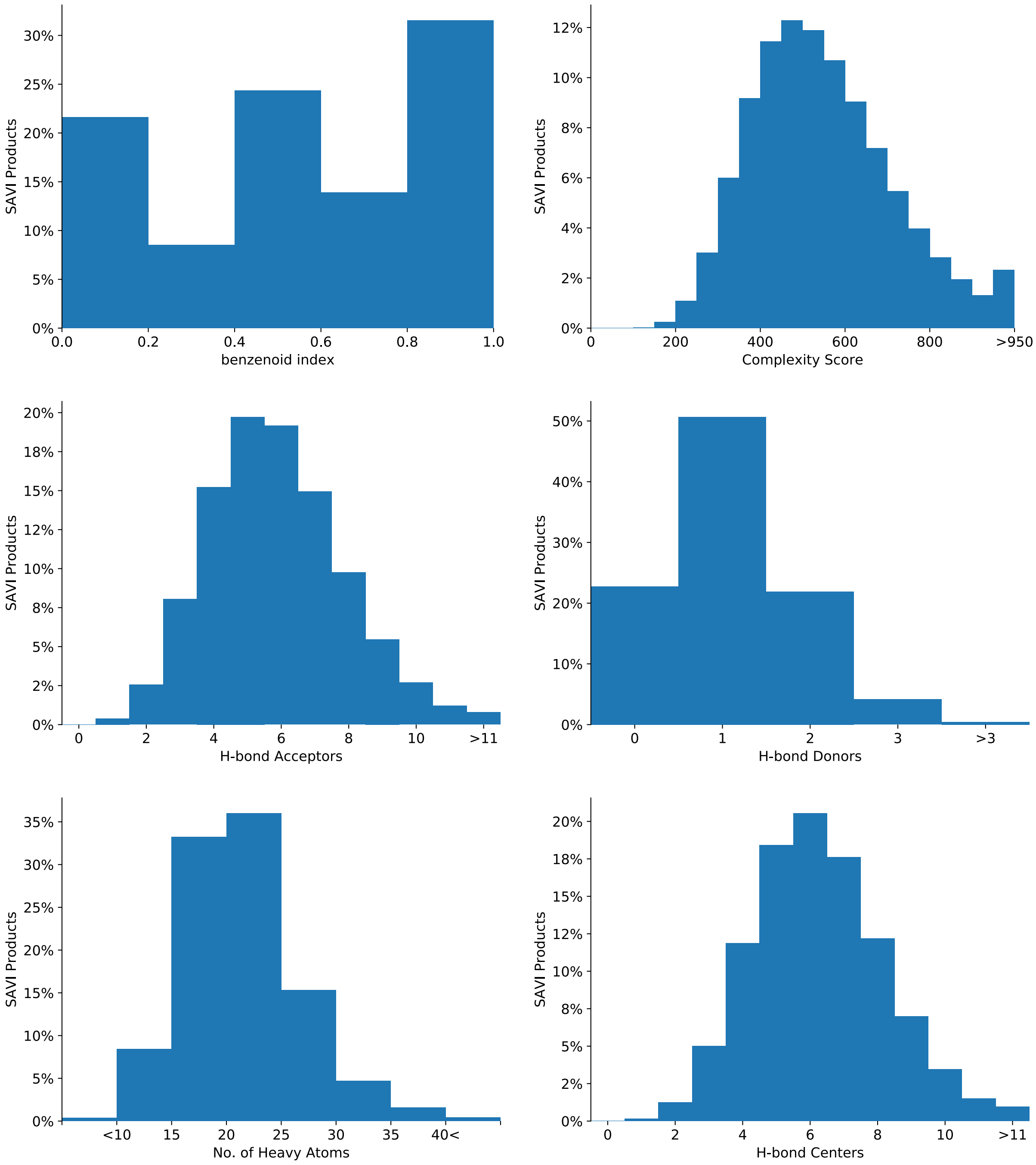

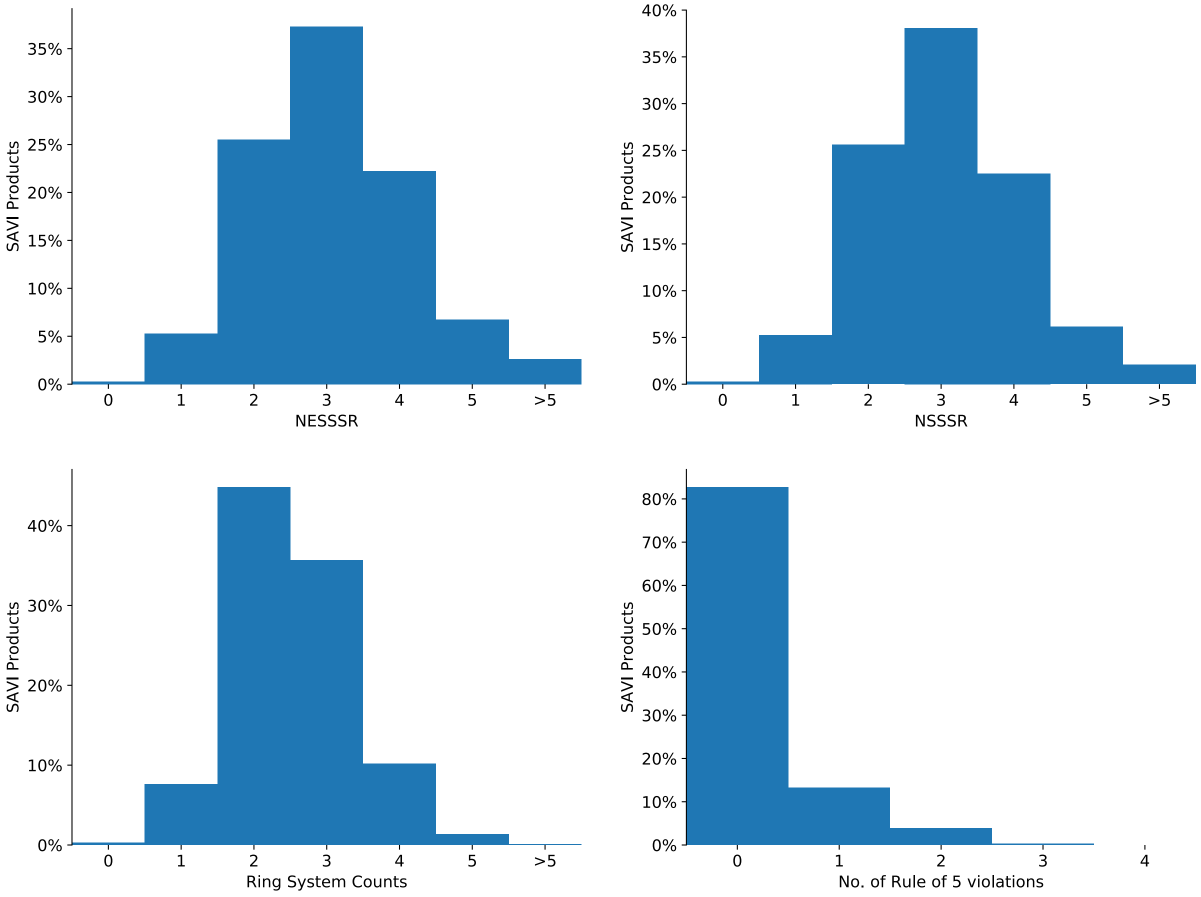
SAVI Products
35%
30%
25%
20%
15%
10%
5%
0%
0
1
2
3
4
5
>5
NESSSR
40%
35%
30%
25%
20%
15%
10%
5%
0%
NSSSR
40%
30%
20%
10%
0%
Ring System Counts
80%
70%
60%
50%
40%
30%
20%
10%
0%
No. of Rule of 5 violations

5. Plots of property distribution for "Neg0" set

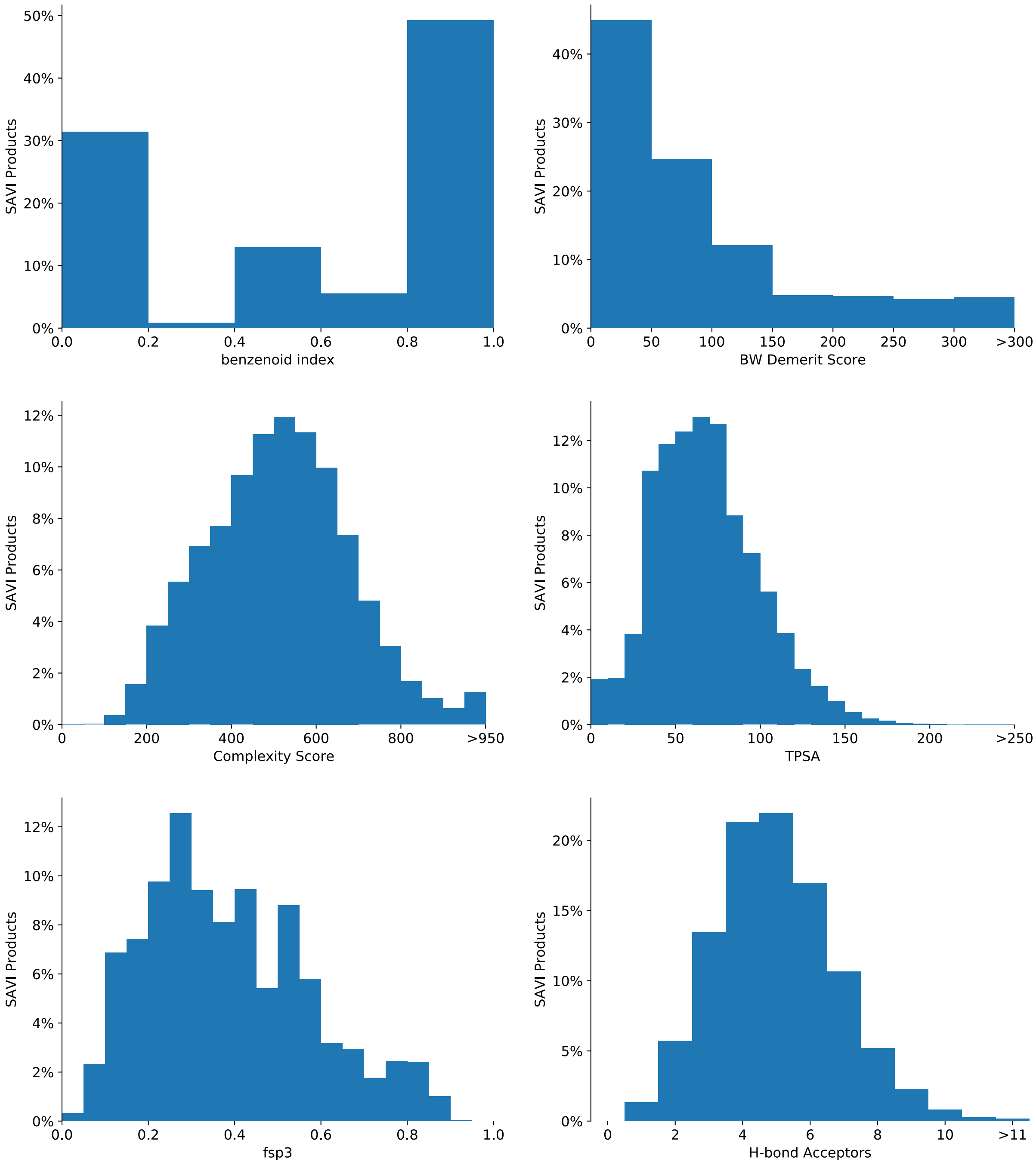

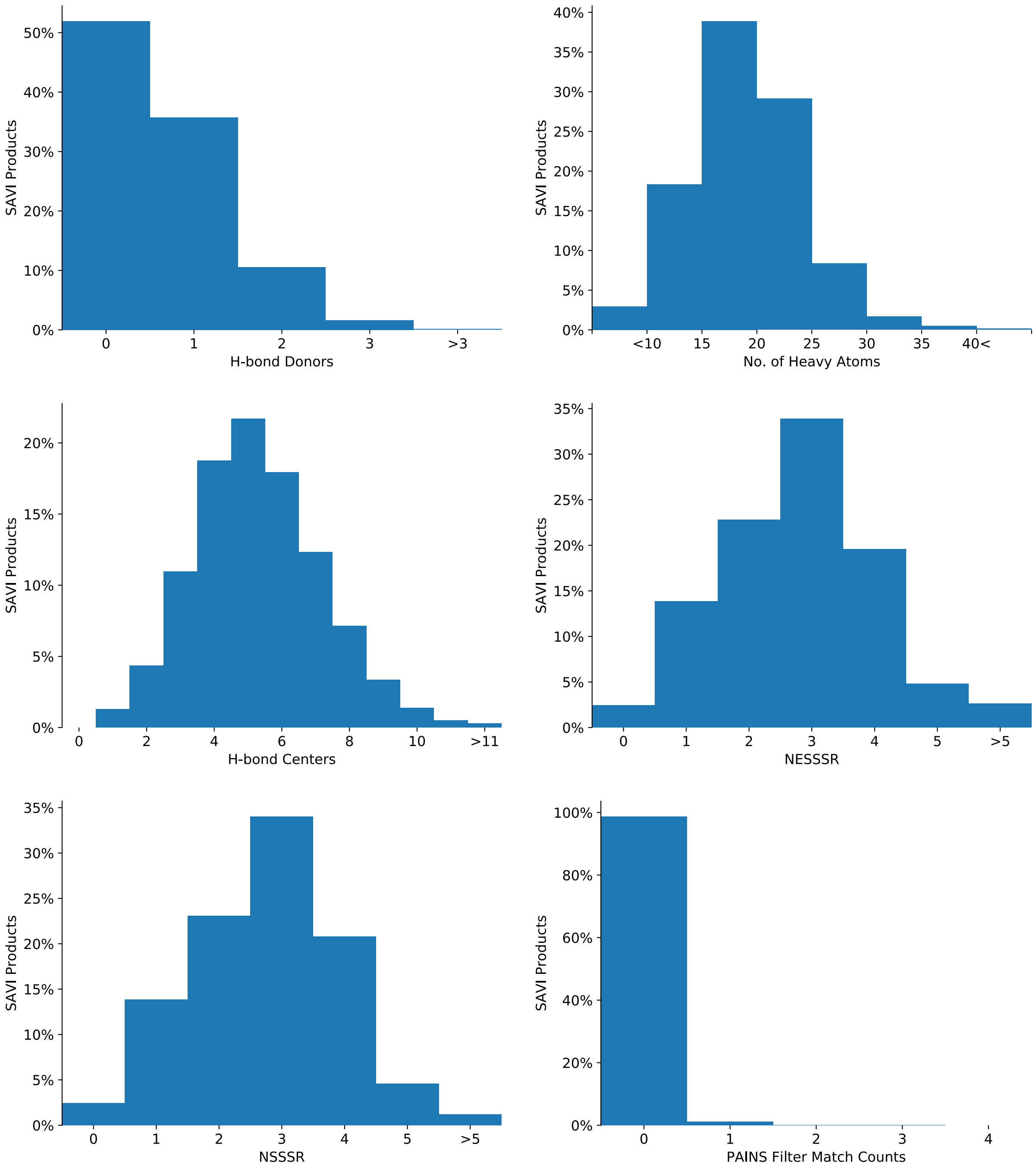
SAVI Products
H-bond Donors
0
1
2
3
>3
0%
10%
20%
30%
40%
50%
No. of Heavy Atoms
<10
15
20
25
30
35
40<
5%
15%
25%
35%
H-bond Centers
4
6
8
10
>11
NESSSR
4
5
>5
NSSSR
PAINS Filter Match Counts
60%
80%
100%

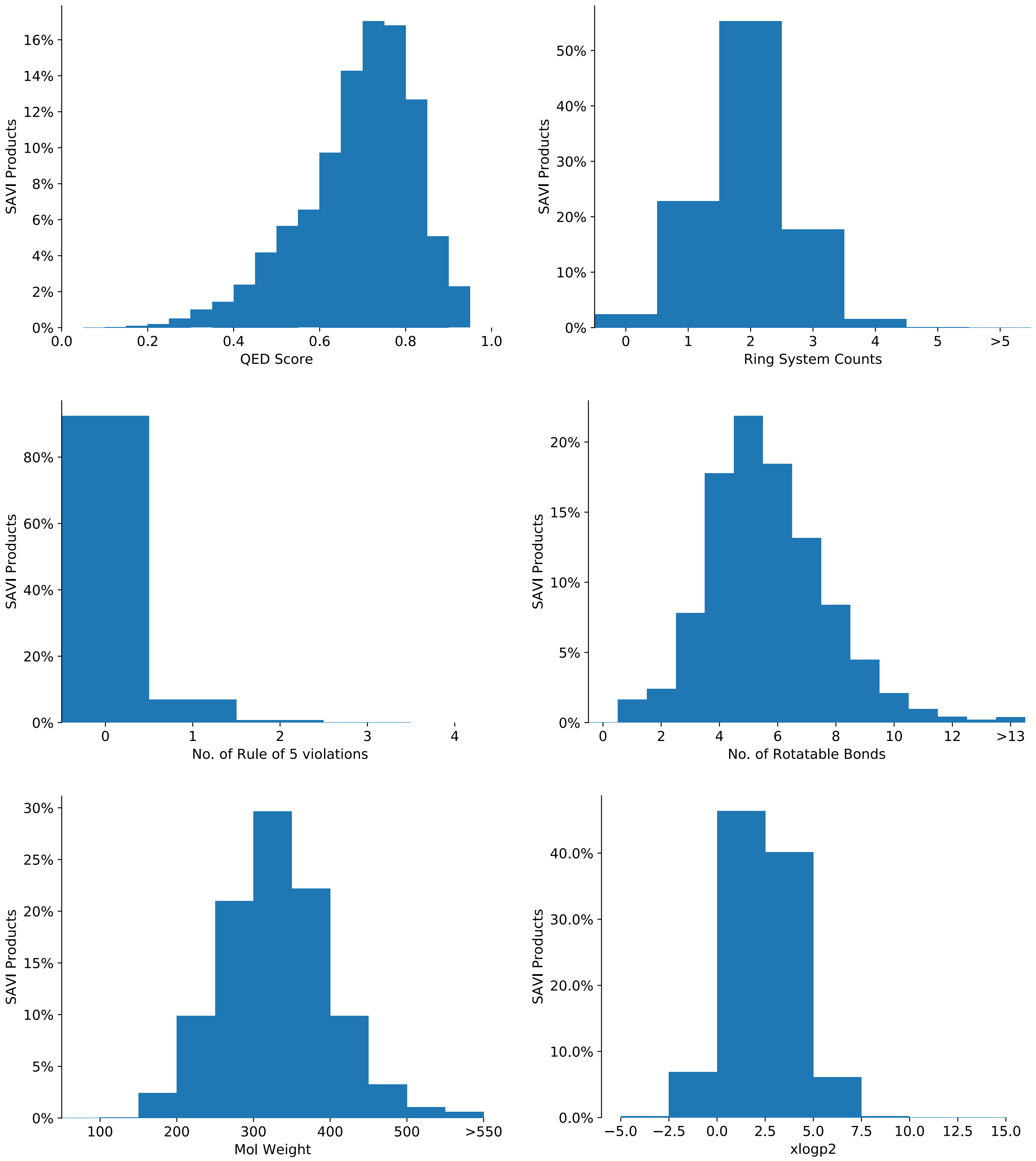
SAVI Products
0% 2% 4% 6% 8% 10% 12% 14% 16%
0.0 0.2 0.4 0.6 0.8 1.0
QED Score
SAVI Products
0% 10% 20% 30% 40% 50%
0 1 2 3 4 5 >5
Ring System Counts
SAVI Products
0% 20% 40% 60% 80%
0 1 2 3 4
No. of Rule of 5 violations
SAVI Products
0% 5% 10% 15% 20%
0 2 4 6 8 10 12 >13
No. of Rotatable Bonds
SAVI Products
0% 5% 10% 15% 20% 25% 30%
100 200 300 400 500 >550
Mol Weight
SAVI Products
0.0% 10.0% 20.0% 30.0% 40.0%
−5.0 −2.5 0.0 2.5 5.0 7.5 10.0 12.5 15.0
xlogp2

6. Plots of property distribution for “Neg10” set

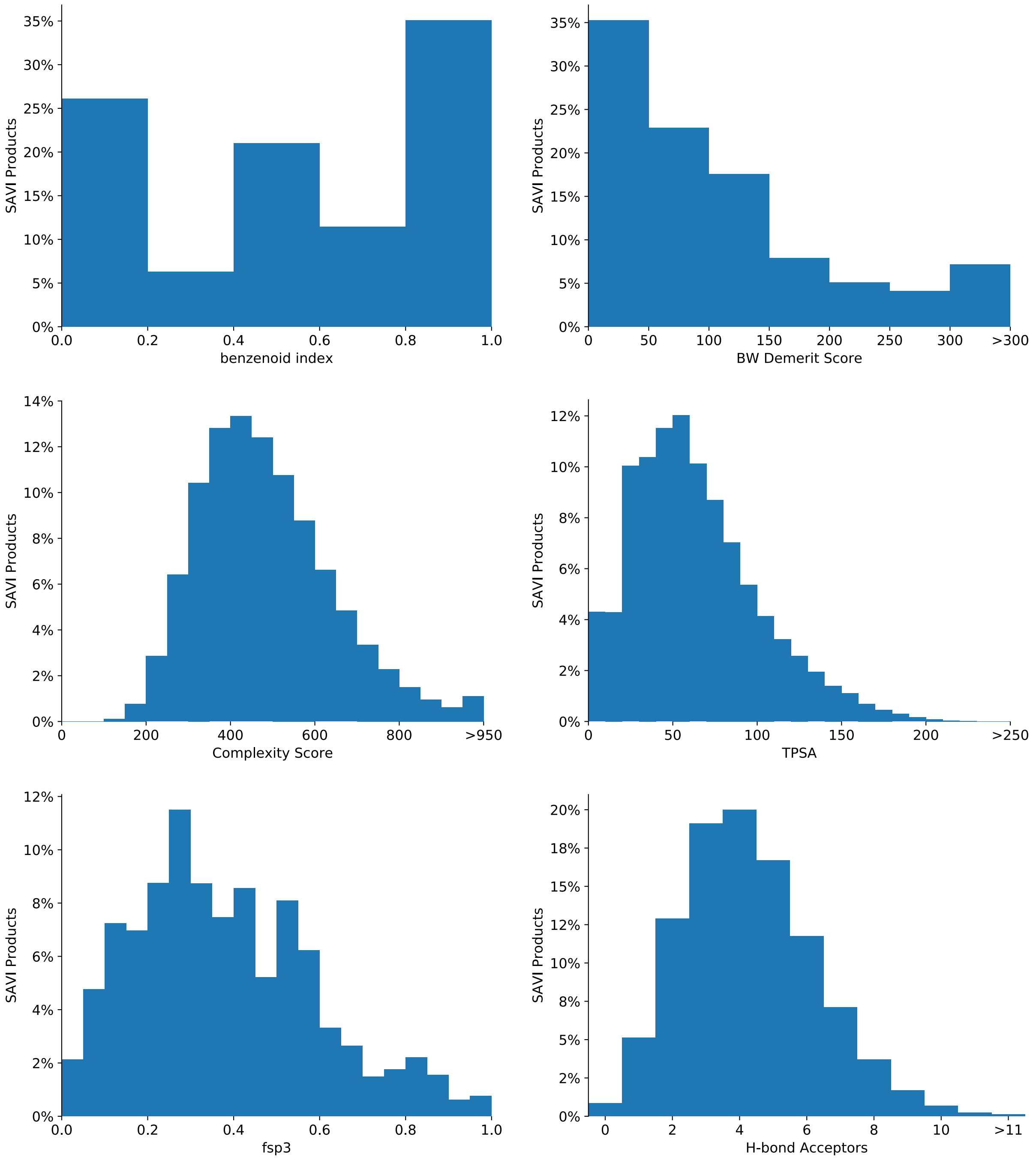

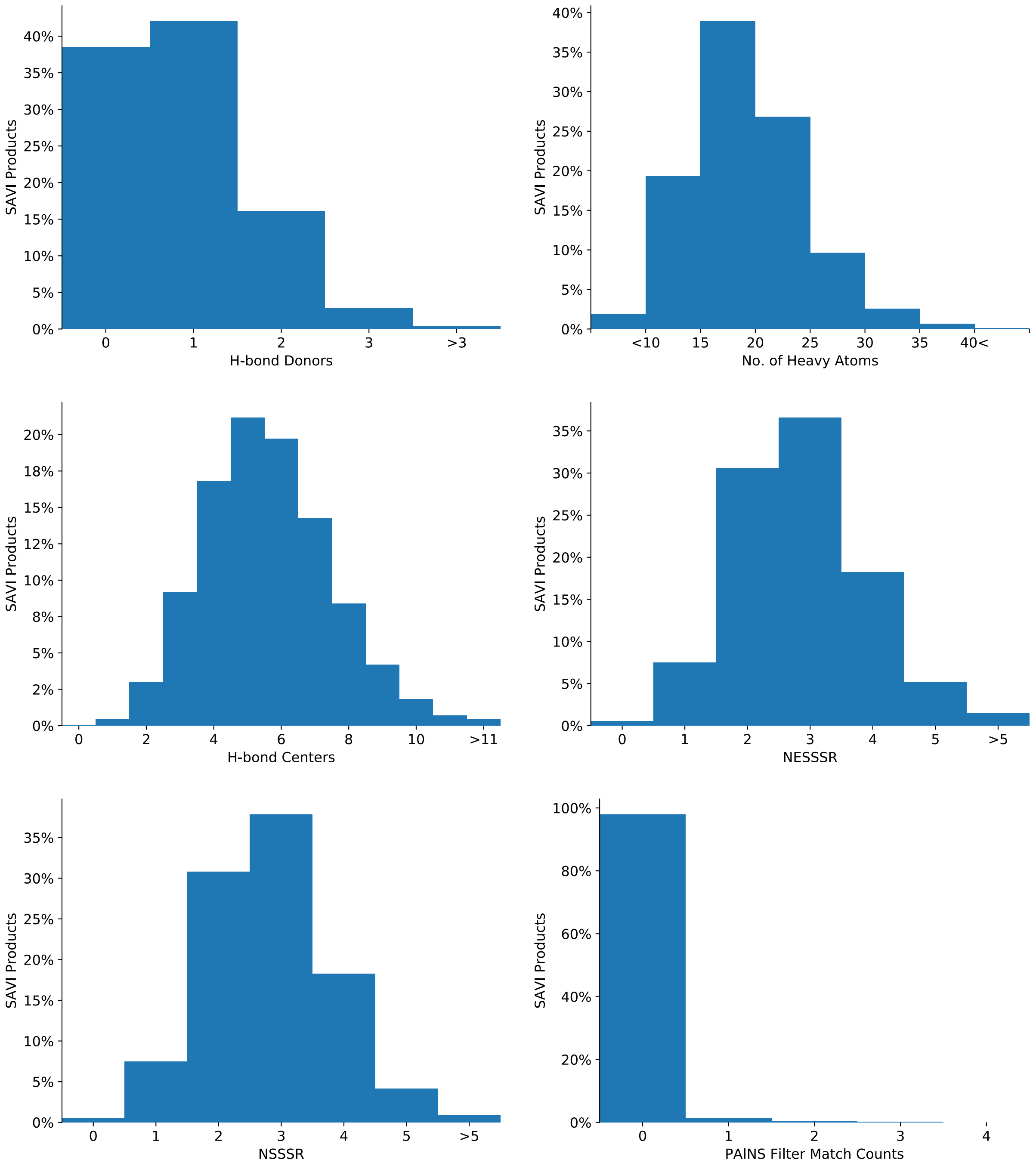
SAVI Products
H-bond Donors
0%
5%
10%
15%
20%
25%
30%
35%
40%
0
1
2
3
>3
SAVI Products
No. of Heavy Atoms
0%
5%
10%
15%
20%
25%
30%
35%
40%
<10
15
20
25
30
35
40<
SAVI Products
H-bond Centers
0%
2%
5%
8%
10%
12%
15%
18%
20%
0
2
4
6
8
10
>11
SAVI Products
NESSSR
0%
5%
10%
15%
20%
25%
30%
35%
0
1
2
3
4
5
>5
SAVI Products
NSSSR
0%
5%
10%
15%
20%
25%
30%
35%
0
1
2
3
4
5
>5
SAVI Products
PAINS Filter Match Counts
0%
20%
40%
60%
80%
100%
0
1
2
3
4

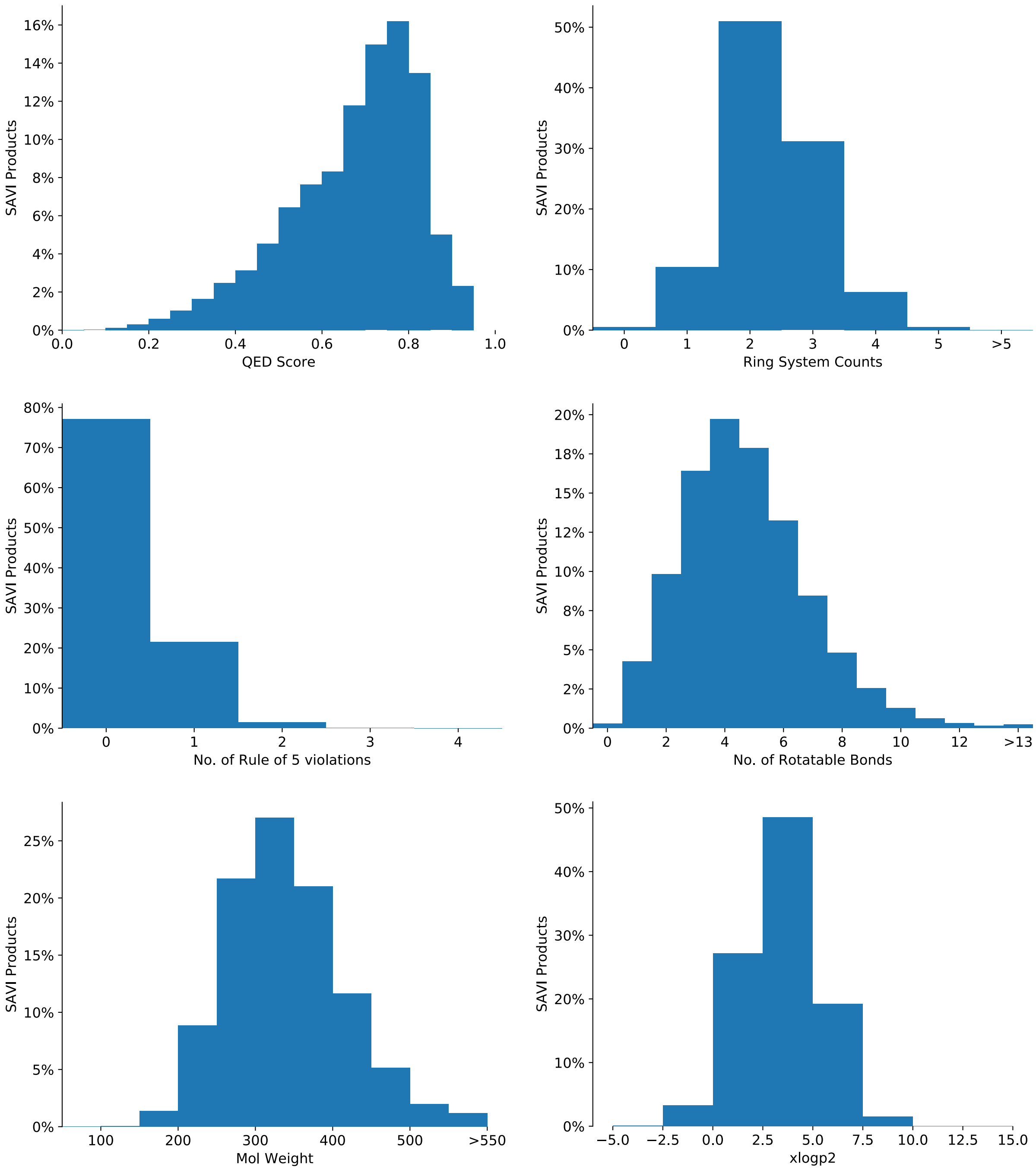
SAVI Products
QED Score
Ring System Counts
No. of Rule of 5 violations
No. of Rotatable Bonds
Mol Weight
xlogp2

7. Plots of property distribution for “Neg20” set

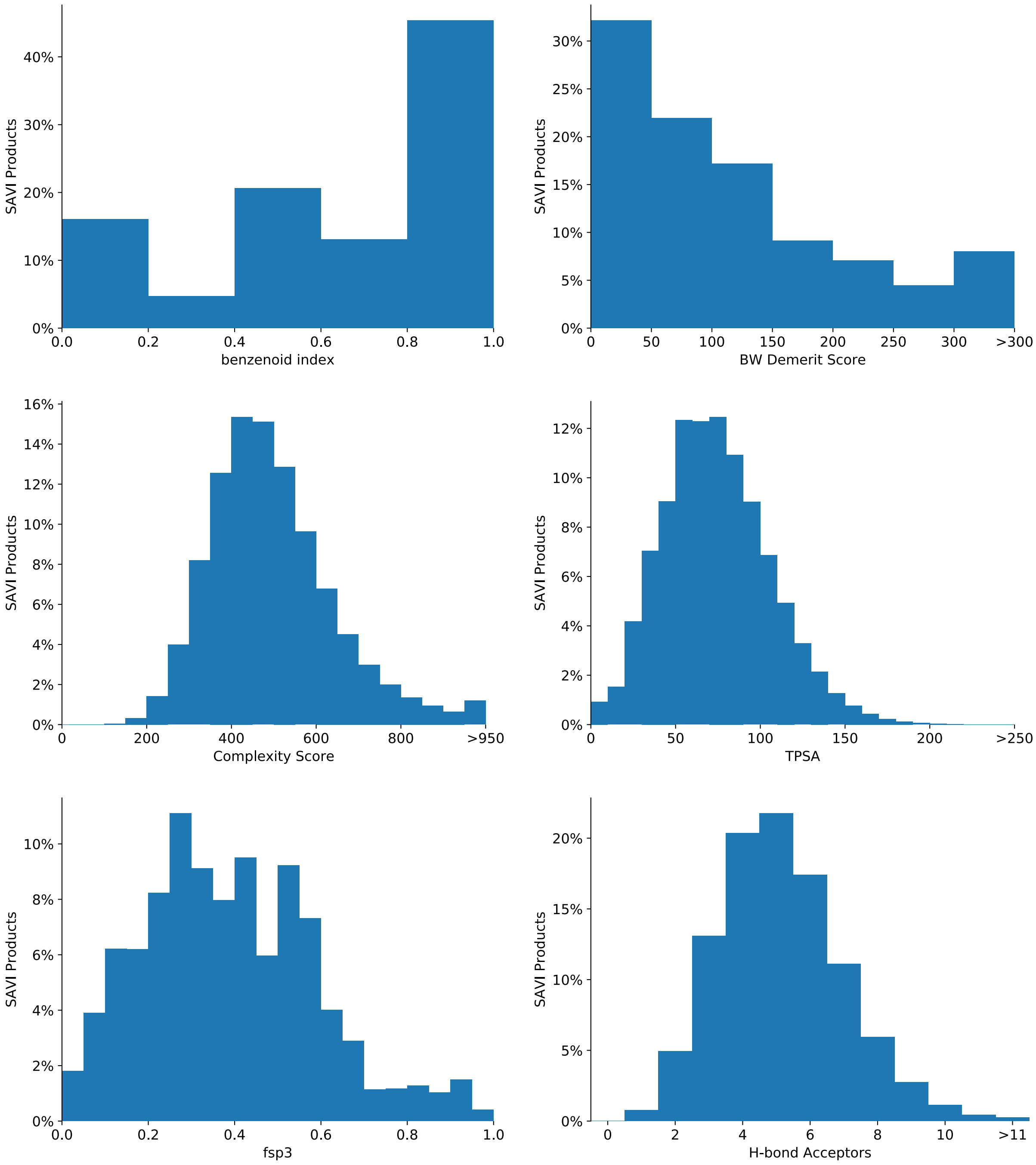

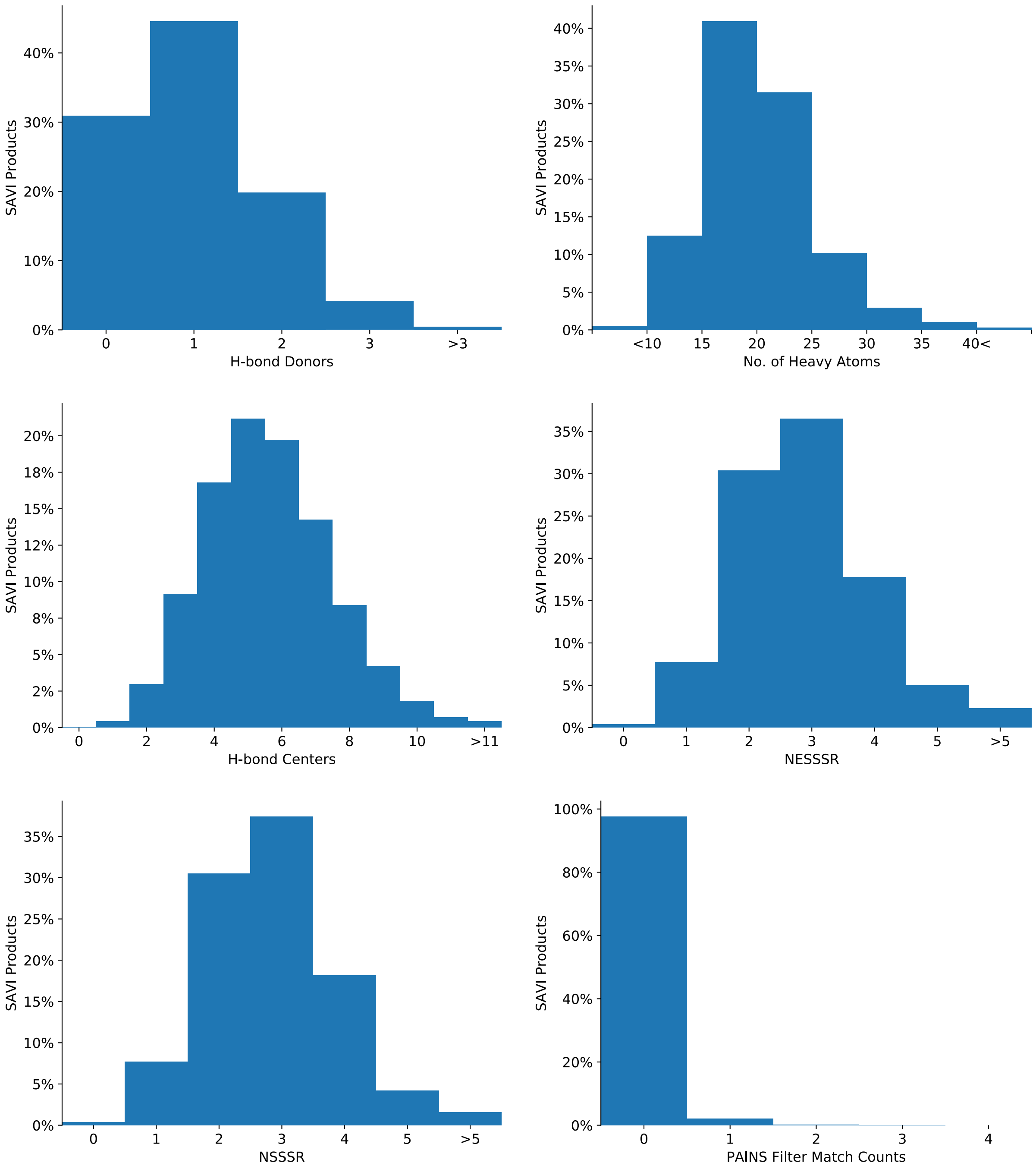
SAVI Products
H-bond Donors
0
1
2
3
>3
0%
10%
20%
30%
40%
No. of Heavy Atoms
<10
15
20
25
30
35
40<
5%
15%
25%
35%
H-bond Centers
4
6
8
10
>11
2%
8%
12%
18%
NESSSR
>5
NSSSR
PAINS Filter Match Counts
60%
80%
100%

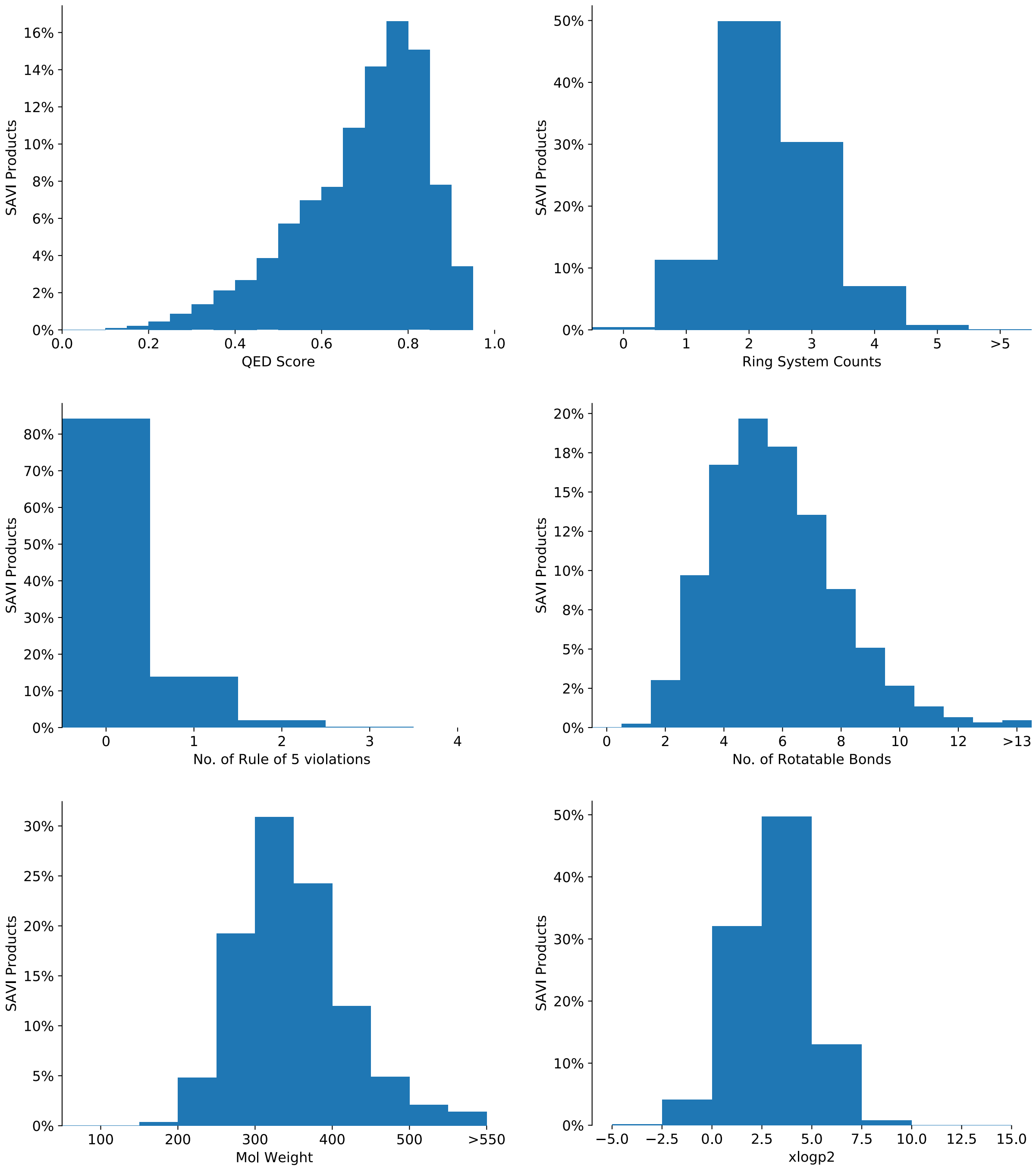
SAVI Products
16%
14%
12%
10%
8%
6%
4%
2%
0%
0.0
0.2
0.4
0.6
0.8
1.0
QED Score
SAVI Products
50%
40%
30%
20%
10%
0%
0
1
2
3
4
5
>5
Ring System Counts
SAVI Products
80%
70%
60%
50%
40%
30%
20%
10%
0%
0
1
2
3
4
No. of Rule of 5 violations
SAVI Products
20%
18%
15%
12%
10%
8%
5%
2%
0%
0
2
4
6
8
10
12
>13
No. of Rotatable Bonds
SAVI Products
30%
25%
20%
15%
10%
5%
0%
100
200
300
400
500
>550
Mol Weight
SAVI Products
50%
40%
30%
20%
10%
0%
−5.0
−2.5
0.0
2.5
5.0
7.5
10.0
12.5
15.0
xlogp2

8. Plots of property distribution for “Neg30” set

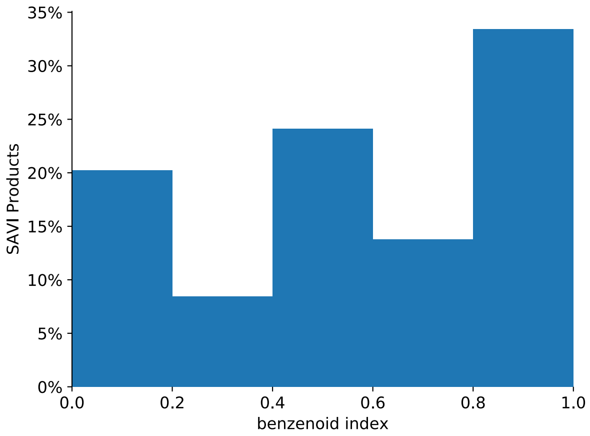

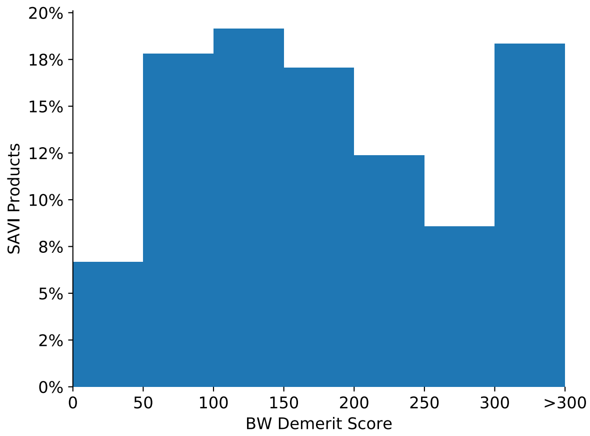

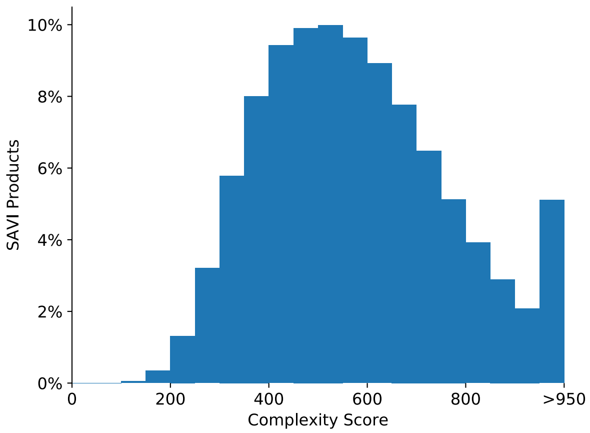

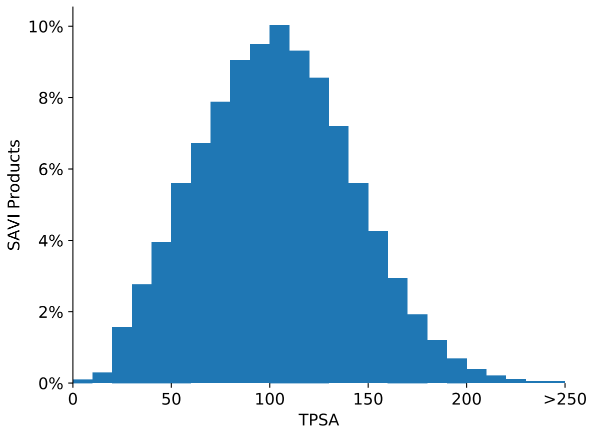

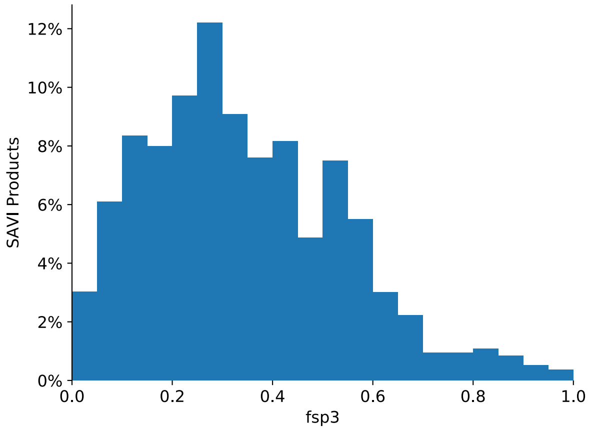

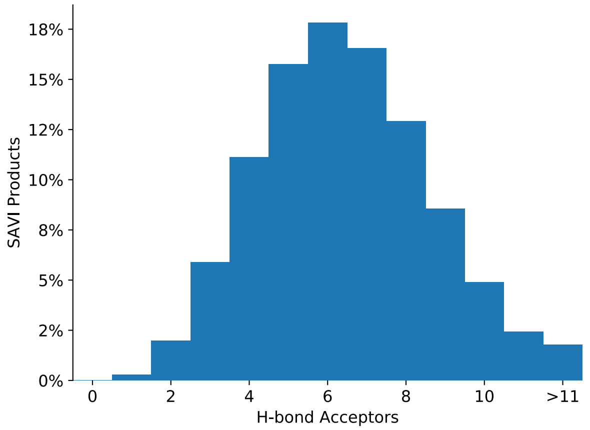

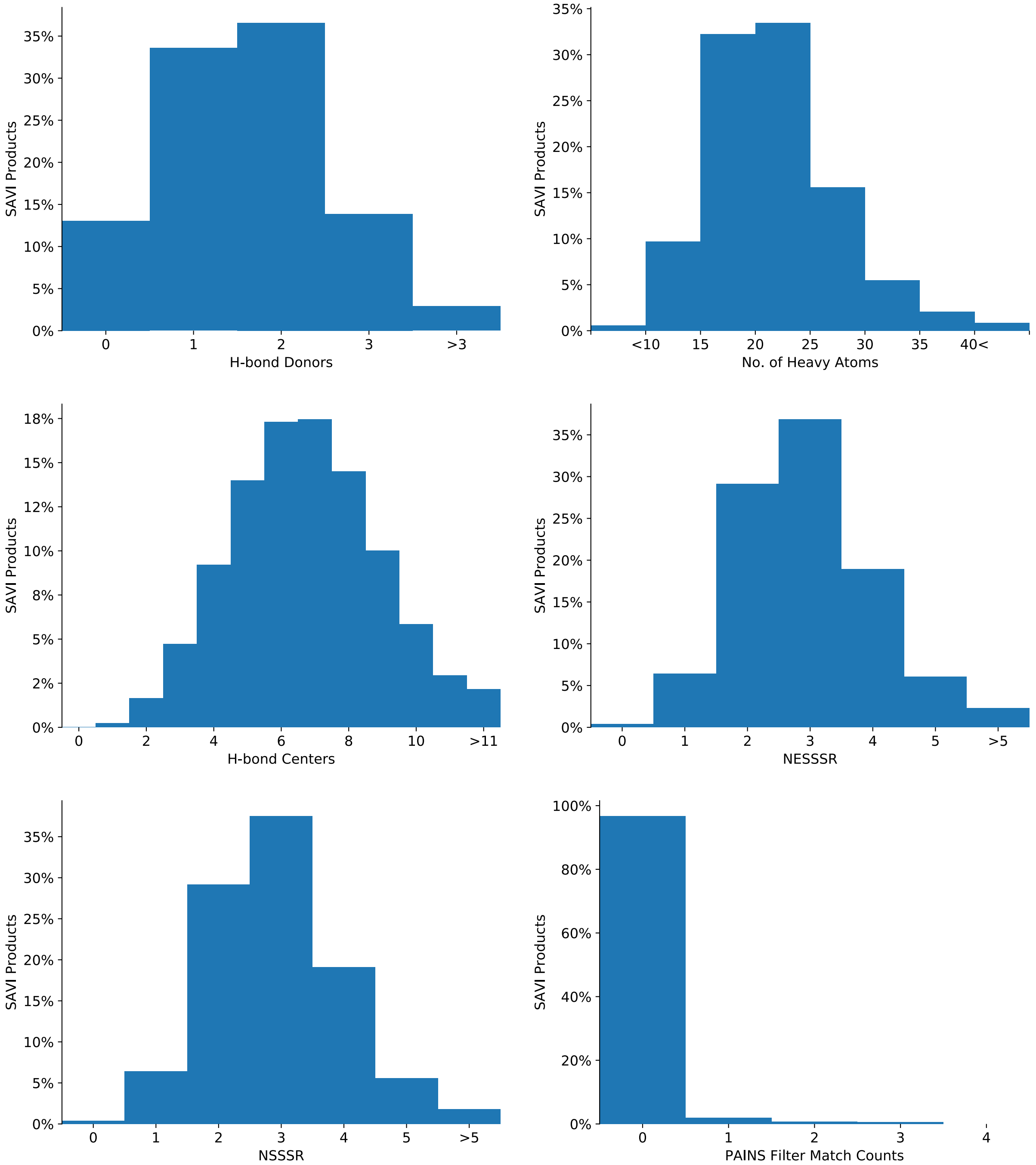
SAVI Products
H-bond Donors
No. of Heavy Atoms
H-bond Centers
NESSSR
NSSSR
PAINS Filter Match Counts
0% 5% 10% 15% 20% 25% 30% 35%
0 1 2 3 >3
<10 15 20 25 30 35 40<
0% 2% 5% 8% 10% 12% 15% 18%
0 2 4 6 8 10 >11
0 1 2 3 4 5 >5
0% 20% 40% 60% 80% 100%
0 1 2 3 4

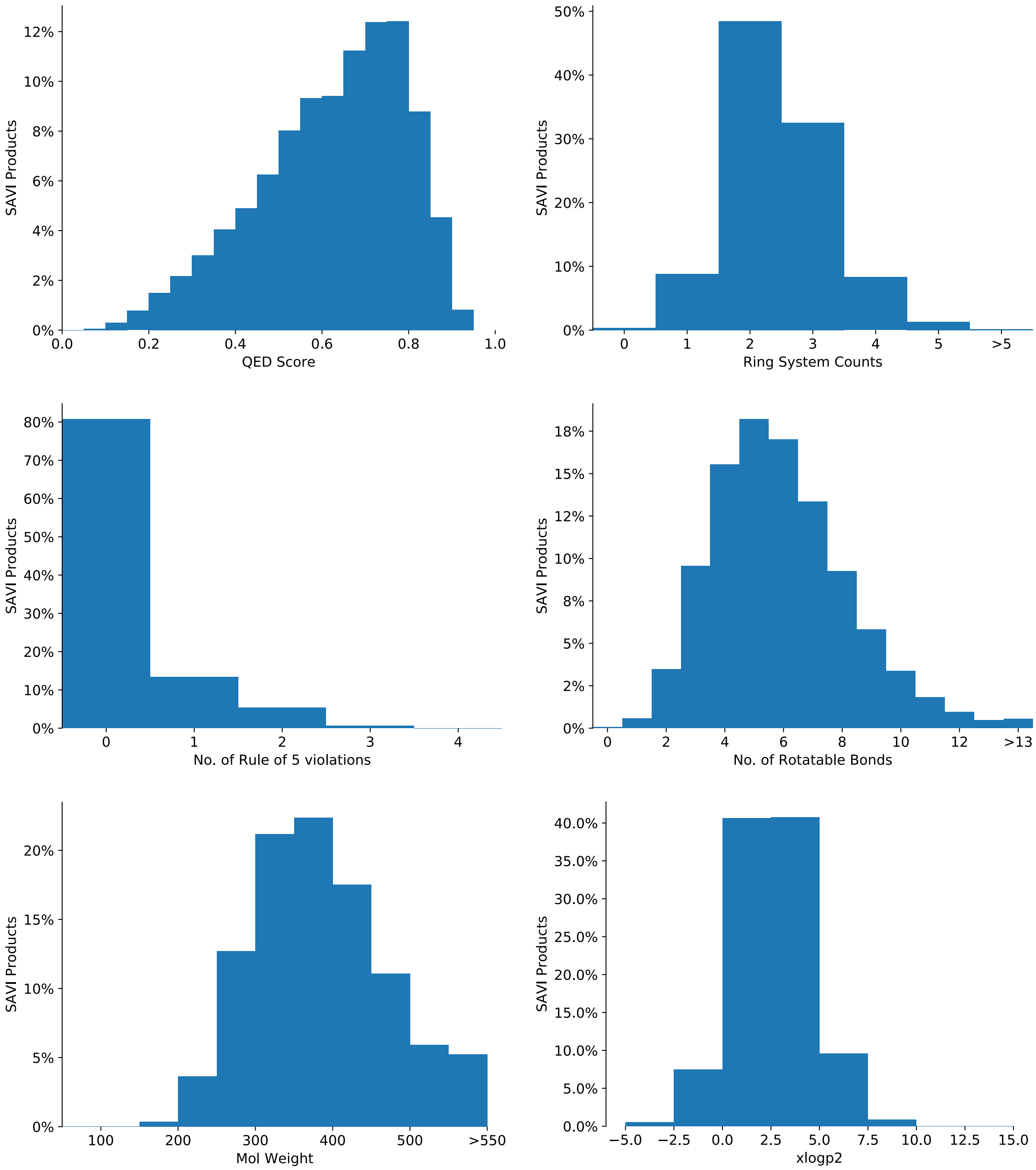
SAVI Products
QED Score
0.0 0.2 0.4 0.6 0.8 1.0
0% 2% 4% 6% 8% 10% 12%
SAVI Products
Ring System Counts
0 1 2 3 4 5 >5
0% 10% 20% 30% 40% 50%
SAVI Products
No. of Rule of 5 violations
0 1 2 3 4
0% 10% 20% 30% 40% 50% 60% 70% 80%
SAVI Products
No. of Rotatable Bonds
0 2 4 6 8 10 12 >13
0% 2% 5% 8% 10% 12% 15% 18%
SAVI Products
Mol Weight
100 200 300 400 500 >550
0% 5% 10% 15% 20%
SAVI Products
xlogp2
−5.0 −2.5 0.0 2.5 5.0 7.5 10.0 12.5 15.0
0.0% 5.0% 10.0% 15.0% 20.0% 25.0% 30.0% 35.0% 40.0%
